# Transcription factor–driven alternative localization of *Cryptococcus neoformans* superoxide dismutase

**DOI:** 10.1016/j.jbc.2021.100391

**Published:** 2021-02-07

**Authors:** Aaron D. Smith, Sarela Garcia-Santamarina, Martina Ralle, David R. Loiselle, Timothy A. Haystead, Dennis J. Thiele

**Affiliations:** 1 Department of Pharmacology and Cancer Biology, Duke University, Durham, North Carolina, USA; 2 Department of Molecular and Medical Genetics, Oregon Health and Science University, Portland, Oregon, USA; 3 Department of Biochemistry, Duke University, Durham, North Carolina, USA; 4 Department of Molecular Genetics and Microbiology, Duke University, Durham, North Carolina, USA

**Keywords:** *Cryptococcus neoformans*, superoxide dismutase, copper, transcription factor, gene regulation, mRNA-sequencing, ChIP-sequencing, subcellular fractionation, posttranslational modification, infection, 5’-RACE, 5’-rapid amplification of cDNA ends, 5’-UTR, 5’-untranslated region, AOX, alternative oxidase, BCS, bathocuproinedisulfonic acid, CFU, colony forming unit, CTR, copper transporter, Cu, copper, CuRE, copper responsive element, HA, hemagglutinin, ICP-MS, inductively coupled plasma mass spectrometry, IMS, intermembrane space, MIP, mitochondrial import peptide, MT, metallothionein, NAT, N-acetyltransferase, ROS, reactive oxygen species, SC, synthetic complete, SOD, superoxide dismutase

## Abstract

*Cryptococcus neoformans* is an opportunistic fungal pathogen whose pathogenic lifestyle is linked to its ability to cope with fluctuating levels of copper (Cu), an essential metal involved in multiple virulence mechanisms, within distinct host niches. During lethal cryptococcal meningitis in the brain, *C. neoformans* senses a Cu-deficient environment and is highly dependent on its ability to scavenge trace levels of Cu from its host and adapt to Cu scarcity to successfully colonize this niche. In this study, we demonstrate for this critical adaptation, the Cu-sensing transcription factor Cuf1 differentially regulates the expression of the *SOD1* and *SOD2* superoxide dismutases in novel ways. Genetic and transcriptional analysis reveals Cuf1 specifies 5’-truncations of the *SOD1* and *SOD2* mRNAs through specific binding to Cu responsive elements within their respective promoter regions. This results in Cuf1-dependent repression of the highly abundant *SOD1* and simultaneously induces expression of two isoforms of *SOD2*, the canonical mitochondrial targeted isoform and a novel alternative cytosolic isoform, from a single alternative transcript produced specifically under Cu limitation. The generation of cytosolic Sod2 during Cu limitation is required to maintain cellular antioxidant defense against superoxide stress both *in vitro* and *in vivo*. Further, decoupling Cuf1 regulation of Sod2 localization compromises the ability of *C. neoformans* to colonize organs in murine models of cryptococcosis. Our results provide a link between transcription factor–mediated alteration of protein localization and cell proliferation under stress, which could impact tissue colonization by a fungal pathogen.

*Cryptococcus neoformans* is a debilitating opportunistic fungal pathogen responsible for nearly 250,000 deaths per year worldwide (1). *C. neoformans* primarily impacts immune-compromised individuals such as those receiving immunosuppressive therapies (cancer, diabetes, and organ transplant patients) or have underlying immune deficiencies (HIV/AIDS), resulting in high mortality rates (40–80%) (1, 2). More recently, a closely related *Cryptococcus gattii* strain emerged that was responsible for the infectious outbreak in the Pacific northwest region of the United States of America and Canada, which also infected immune-competent individuals (3, 4, 5). As a successful human pathogen, *C. neoformans* must adapt to environmental extremes and insults encountered within the host during infection and is reliant upon the host for nutrients and essential trace metals (6, 7). However, through a process termed nutritional immunity, the immune system actively sequesters and withholds nutritional Fe, Mn, and Zn from pathogens to deprive them of essential trace elements (8). In turn, successful pathogens have evolved sophisticated mechanisms to acquire metals from their host, circumventing nutritional immunity. In contrast to Fe, Mn, and Zn limitation, the host elevates levels of copper (Cu) to induce Cu poisoning within the hostile environment of macrophage phagosomes (9, 10, 11, 12, 13). Additionally, in response to bacterial urinary tract infections, high levels of antimicrobial Cu is secreted into the urine (14, 15, 16).

Cu has potent antimicrobial activities and, in part, exerts its effects through uncontrolled redox cycling to propagate the formation of toxic reactive oxygen species (ROS) (17, 18). More recent work has shown that Cu also exerts toxicity through displacement of Fe from essential Fe-S cluster containing enzymes, rendering them inactive, and by disrupting the Fe-S cluster biogenesis machinery (19, 20, 21, 22). However, local Cu concentrations also change in response to microbial infection in a tissue-specific manner. For example, Cu levels decrease in the kidney during chronic infection by *Candida albicans* (23, 24). Moreover, while whole animal imaging demonstrated that *C. neoformans* is exposed to elevated host Cu levels mobilized in alveolar macrophages, it senses Cu deficiency in the brain during cryptococcal meningitis and responds by activating expression of the Cu^+^ import machinery (25, 26). It has also been reported that Cu deprivation can occur within macrophages, in a manner similar to traditional metal withholding strategies, suggesting immune responses may vary dependent upon infection type, stimulation of different immune response pathways, or acute *versus* chronic stages of infection (27, 28). It is yet to be determined whether decreases in local Cu concentrations are because of inherent low bioavailability of Cu within these host niches or if *bona fide* Cu nutritional immunity mechanisms are implemented in response to infection, similar to those described for Fe, Mn, and Zn. Interestingly, Cu has also been shown to promote *Helicobacter pylori* colonization of gut epithelia through interactions with host trefoil peptides (29). Thus, Cu has many facets mediating a dynamic interplay between host–pathogen interactions and pathogenic outcomes during infection.

Cu catalyzes numerous biochemical functions, many of which are required for virulence in *C. neoformans* (6, 30). Cu serves as a cofactor for cellular respiration, high affinity iron uptake, melanin production, as well as detoxification of superoxide radical anions *via* superoxide dismutase (SOD), among other functions (31, 32, 33, 34). Traditionally, SOD enzymes are critical cellular antioxidants that are evolutionarily highly conserved in structure and function and catalyze the disproportionation of superoxide into O_2_ and H_2_O_2_ (35, 36). Eukaryotes typically express at least two classes of SODs, those that require Cu and Zn for catalysis (Sod1) and those that use Mn as a catalytic cofactor (Sod2). In Cu, Zn Sod1 enzymes, the Zn cofactor serves solely a structural role in maintaining the active site, whereas the Cu cofactor is the catalytic redox center (37). The mature Sod1 enzyme is a homodimer and is primarily localized within the cytoplasm, with a small but significant fraction also localized within the mitochondrial intermembrane space (IMS) (38). Sod2, a Mn-dependent enzyme, catalyzes the identical chemistry for superoxide anion disproportionation as Sod1. Sod2 is targeted to the mitochondrial matrix by an encoded amino-terminal mitochondrial import peptide (MIP). This positively charged leader sequence is recognized by the mitochondrial import machinery (TOM/TIM complexes), and Sod2 is translocated into the mitochondria as the nascent peptide exits the ribosome in an unfolded or semiunfolded state (39, 40). As Sod2 is transported into the mitochondrial lumen, an inner membrane peptidase cleaves the MIP, and with the help of the Mtm1 inner membrane chaperone, Sod2 is thought to fold around its Mn cofactor (37, 41). The mature form of Sod2 then assembles as an active homotetramer in the mitochondrial lumen.

Pathogens must secure sufficient quantities of Cu, Zn, and Mn from their hosts to sustain SOD activity, a daunting challenge in the face of host imposed nutritional immunity. Here, we demonstrate that the Cu-sensing transcription factor Cuf1 orchestrates an adaptive response to Cu limitation through both the reciprocal regulation of the SODs, *SOD1* and *SOD2*, and by driving a change in the subcellular location of Sod2. Cuf1 represses expression of the highly abundant *SOD1* and simultaneously induces expression of two isoforms of *SOD2* from an alternative transcript produced specifically under Cu limitation: the canonical mitochondrial targeted isoform and a novel alternative cytosolic isoform. Cuf1 drives the generation of *SOD1* and *SOD2* transcripts that have shorter 5’ ends, as compared with those species found under Cu sufficiency conditions, through recognition of Cu-responsive elements (CuREs) within their promoter regions. Replacement of Sod1 with cytosolic Sod2 is required to maintain cellular superoxide buffering and contributes to the ability of *C. neoformans* to resist oxidative stress and impacts colonization of the brain during cryptococcal meningitis. Thus, Cuf1 plays a vital role in virulence by activating the Cu detoxification machinery in the lung, the Cu uptake machinery in the brain, and by altering the subcellular localization of a Mn-dependent Sod2 protein that is required for protection from the oxidative burst.

## Results

### Cuf1 reciprocally regulates SOD1 and SOD2 during Cu limitation

**Figure 1:**
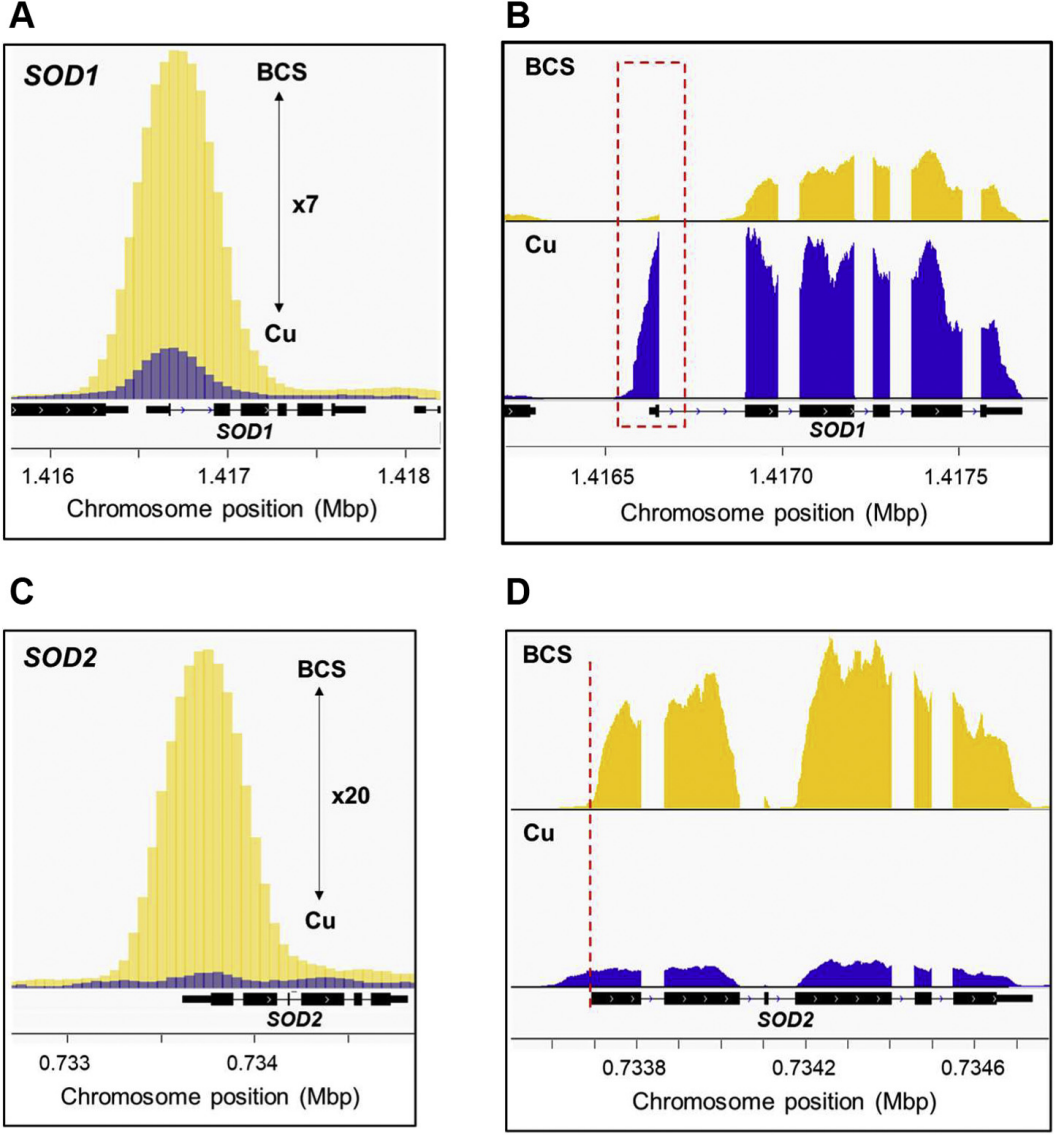
**ChIP-seq and RNA-seq analysis of *C. neoformans* grown during Cu deficiency demonstrates differential regulation of *SOD1* and *SOD2***. *A*, ChIP-seq analysis indicates Cuf1 is enriched in the *SOD1* (CNAG_01019) promoter during Cu deficiency (*yellow*) *versus* Cu excess (*blue*). Sequencing reads were aligned to the *C. neoformans* H99 genome and visualized using the integrative genome viewer (IGV) (84). *B*, mRNA-seq analysis shows a 3-fold induction of *SOD1* transcript abundance in Cu excess (*blue*) *versus* Cu deficiency (*yellow*) conditions. *SOD1* transcripts with shortened 5’-ends generated during Cu limitation which omit the first coding exon are indicated by the *red box*. *C*, ChIP-seq analysis of the *SOD2* (CNAG_04388) promoter as in (*A*). *D*, mRNA-seq analysis of *SOD2* as in (*B*). *SOD2* transcript with shortened 5’-ends indicated by the *red dashed line*. X-axis representative of chromosomal location (in Mbp) of *SOD1* and *SOD2*. BCS, bathocuproinedisulfonic acid; SOD, superoxide dismutase.

From our previous comparative mRNA-seq and ChIP-seq analysis of *C. neoformans* cultures grown under elevated Cu or Cu limiting conditions, the genome-wide Cu-responsive, Cuf1-dependent regulon of *C. neoformans* was identified (42). This work revealed 96 genes in the Cuf1 regulon, of which two encode the Cu, Zn-dependent *SOD1* (CNAG_01019) and the Mn-dependent *SOD2* (CNAG_04388). Multiple sequence alignment reveals the conserved nature of the SODs as well as their highly conserved metal coordinating ligands within their respective active site (Fig. S1, *A* and *B*). ChIP-seq data demonstrate strong enrichment of Cuf1 at the *SOD1* promoter primarily during Cu limitation, with a modest enrichment in the presence of Cu (Fig. 1*A*), and mRNA-seq data show a ∼3-fold decrease in *SOD1* transcript levels when cells were supplemented with the extracellular Cu^+^ chelator bathocuproinedisulfonic acid (BCS) (Fig. 1*B*). A strong enrichment of Cuf1 was also observed within the *SOD2* promoter with a ∼6-fold induction in transcript levels when cells were grown under Cu limitation (Fig. 1, *C* and *D*). Further, the regulation of both genes was Cuf1-dependent (Fig. S2, *A* and *B*).

Interestingly, upon inspection of the transcript sequencing reads aligned to the annotated *C. neoformans* H99 genome, a 5’-truncated *SOD1* transcript was almost exclusively detected from Cu-limited cells. This transcript skips the first coding exon 1 (Fig. 1*B*), and its generation is dependent on the presence of Cuf1 (Fig. S2*C*). Additionally, examination of the aligned sequencing reads for *SOD2* revealed that this transcript has a shorter 5’-upstream region when cells were grown under Cu limitation (Fig. 1*D*), the generation of which was also dependent on Cuf1 (Fig. S2*D*). However, compared with the *SOD1* full-length transcript only, the 5’-untranslated region (5’-UTR) of the *SOD2* transcript was omitted, leaving the first exon intact.

It is important to note our previous transcriptome studies used 1 mM CuSO_4_ to induce conditions of Cu excess (42). While markers for Cu excess such as the metallothionein, *MT1* and *MT2*, transcripts are highly induced at this concentration, we also noted several other stress markers that were induced in a non-Cuf1–dependent manner, suggesting activation of Cuf1-independent stress pathways. For the studies described here, we selected 0.1 to 0.3 mM CuSO_4_ for Cu excess, a condition amenable for sustained growth of *C. neoformans*, does not interfere with subsequent biochemical assays, and still highly activates *MT1*, a hallmark for Cu excess (Fig. S2*E*) (43). For several studies, we also used a Cu-sufficient condition of 5 μM CuSO_4_ which neither activated the expression of *MT1* nor the copper transporter *CTR4*, the latter a hallmark of Cu deficiency (Fig. S2, *E* and *F*).

**Figure 2:**
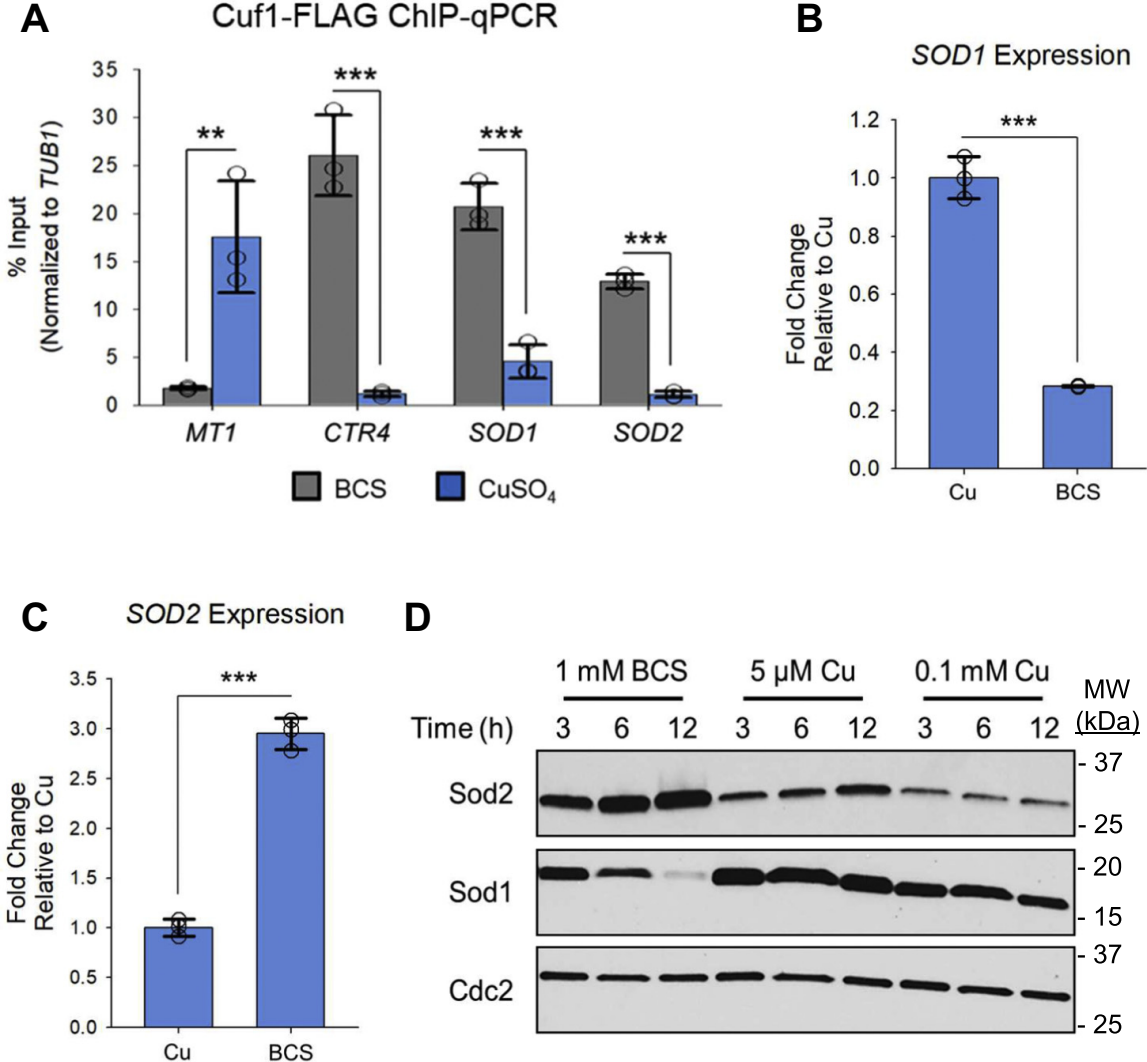
**Cuf1 differentially regulates SOD1 and SOD2 transcription during Cu limitation**. *A*, ChIP-qPCR validation of Cuf1 binding to the *SOD1* and *SOD2* promoters in the *CUF1-FLAG* strain. Cells were treated with 1 mM BCS or 0.3 mM CuSO_4_. ChIP-qPCR of the *MT1* and *CTR4* promoters were included as controls for Cuf1 binding during Cu excess and Cu limitation, respectively. N = 3, 2-tailed *t* test. *B*, qRT-PCR validation of *SOD1* transcript levels. Cells were treated with 1 mM BCS or 0.3 mM CuSO_4_. N = 3, 2-tailed *t* test. *C*, qRT-PCR validation of *SOD2* transcript levels as in (*B*) N = 3, 2-tailed *t* test. *D*, analysis of Sod1 and Sod2 protein expression from *SOD2-HA* cells by Western blotting. Cells were treated with 1 mM BCS or 5 μM CuSO_4_ for the indicated time periods (in hours). Detection of Cdc2 expression was included as a loading control. ∗*p* < 0.05, ∗∗*p* < 0.01, ∗∗∗*p* < 0.005. BCS, bathocuproinedisulfonic acid; SOD, superoxide dismutase.

We further validated the *SOD1* and *SOD2* ChIP-seq (Fig. 2*A*) and mRNA-seq (Fig. 2, *B* and *C*) datasets by ChIP-qPCR and qRT-PCR, respectively. Importantly, we designed our primers to anneal near the 3’-end of the generated *SOD1* and *SOD2* cDNA products to prevent the exclusion of any truncated transcript from the qRT-PCR analysis. To ascertain if there was a correlation between changes in *SOD1* and *SOD2* transcript levels with that of protein abundance, Sod1 and Sod2 protein levels were analyzed from cells cultured in the presence of Cu or BCS. Although an anti-Sod1 antibody against *Saccharomyces cerevisiae* (44) cross-reacted with *C. neoformans* Sod1, there was no cross-reactivity of a yeast anti-Sod2 antibody against *C. neoformans* Sod2 (45). A functional (see below) carboxyl-terminal epitope-tagged allele of *SOD2* (2x-hemagglutinin [HA] epitope at its native genomic locus) was generated for these experiments (*SOD2HA*). In cells grown with sufficient or excess Cu, constitutive expression of Sod1 and Sod2 was detected (Fig. 2*D*). However, cells cultured in the presence of BCS showed a time dependent decrease in Sod1 abundance that paralleled an increase in Sod2 protein levels (Fig. 2*D*).

### Cuf1 binds CuREs within SOD1 and SOD2

**Figure 3:**
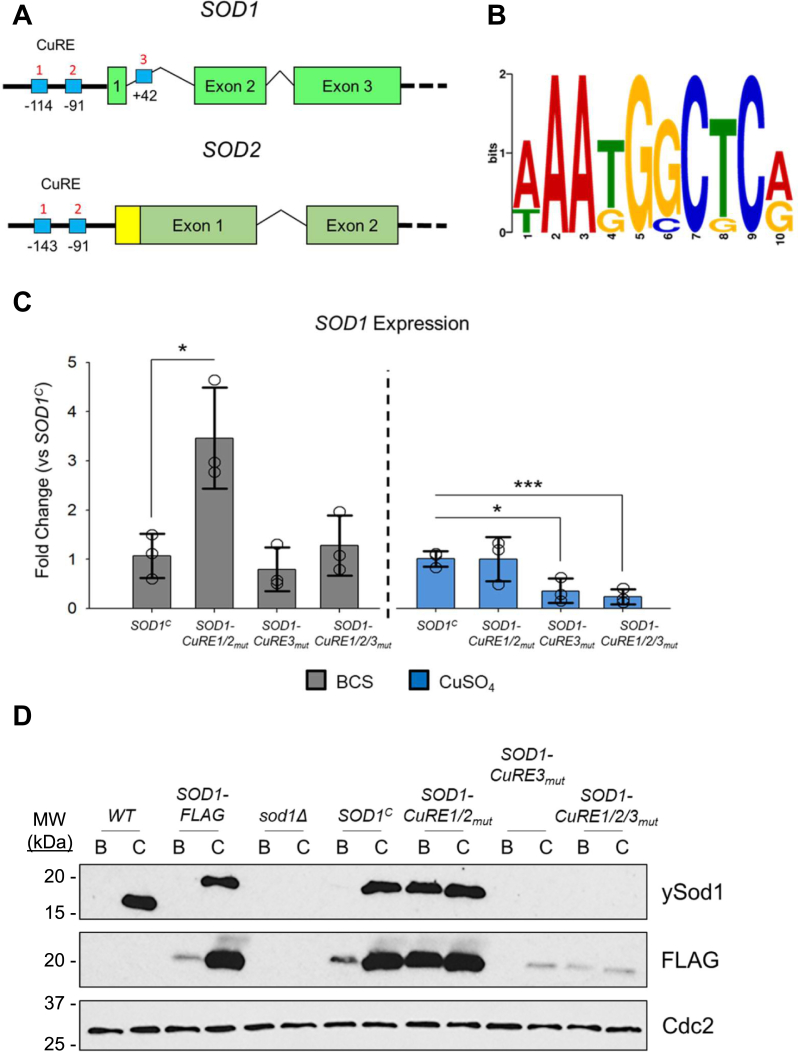
**The CuRE sequences are required for proper regulation of SOD1 during Cu limitation**. *A*, schematic representation of consensus CuRE sequences (*blue boxes*) and number (*red text*) within the promoters of *SOD1* and *SOD2*. *Solid green boxes* represent exons and connecting lines represent introns. The *yellow box* depicts the amino-terminally encoded mitochondrial import peptide (MIP) of *SOD2*. Numbers below CuREs represent start of the sequences relative to the initiating codon (+1). *B*, consensus motif of the CuRE sequences identified using the MEME suite software (46). *C*, qRT-PCR analysis of *SOD1* transcript levels in the indicated strains when cultured with 1 mM BCS (*gray*) or 0.1 mM CuSO_4_ (*blue*). Expression levels were normalized to the *SOD1*^*C*^ strain. N = 3, 2-tailed *t* test. ∗*p* < 0.05, ∗∗*p* < 0.01, ∗∗∗*p* < 0.005. *D*, protein expression levels of Sod1 in the indicated strains treated with 1 mM BCS (*B*) or 0.1 mM CuSO_4_ (*C*) for 12 h. Sod1 was detected using the yeast anti-Sod1 antibody (ySod1) as well as an anti-FLAG epitope antibody (FLAG). Detection of Cdc2 was included as a loading control. CuRE, copper responsive element; SOD, superoxide dismutase.

Cuf1 recognizes and binds to conserved CuREs within the promoter regions of the Cu-deficiency inducible genes (42). Using the MEME suite software (46), putative CuREs embedded in the *SOD1* and *SOD2* genes were identified (Fig. 3*A* and Table S1). For *SOD1*, three putative CuRE sequences were identified, with two located within the 5’-UTR and a single CuRE within the first intron. For *SOD2*, two tandem CuRE sequences within the 5’-UTR were identified. These sequences conform to the consensus 5′-(A/T)AA(T/G)G(G/C)C(T/G)C(A/G)-3′, which closely resembles the CuRE sequences identified in *S. cerevisiae* and *C. albicans* (47, 48, 49) (Fig. 3*B* and Table S1). Unfortunately, because of the large area of Cuf1 enrichment around the *SOD1* and *SOD2* promoters, along with the close proximity of the CuREs to one another, we could not definitively identify which CuREs were involved in mediating Cuf1 binding by our ChIP-seq datasets alone.

To assess the impact of the putative CuREs on *SOD1* expression, a *sod1Δ* strain was generated by replacement of the *SOD1* allele with a neomycin (NEO) cassette. A WT copy of *SOD1* harboring the native promoter and an additional carboxy-terminal FLAG epitope (*SOD1*^*C*^), or an epitope-tagged *SOD1* allele with individually mutated CuRE sites (*SOD1-CuRE1/2*_*mut*_ and *SOD1-CuRE3*_*mut*_) or in combination (*SOD1-CuRE1/2/3*_*mut*_) was re-introduced within the safe haven locus (50) of the *sod1Δ* strain. Additionally, the WT *SOD1* allele was tagged with a carboxyl-terminal FLAG epitope at its native genomic locus (*SOD1FLAG*) to generate an isogenic control strain. The previous qRT-PCR studies revealed that *SOD1* expression is repressed during Cu limitation (Fig. 2*B*), but mutation of CuRE1/2 within the *SOD1* promoter alleviated this repression (Fig. 3*C*). Mutation of CuRE1/2 had no impact on *SOD1* expression in the presence of Cu. The CuRE3 mutant showed a similar extent of repression compared with the *SOD1*^*C*^ strain when treated with BCS but interestingly was also repressed in the presence of Cu (Fig. 3*C*). This repression was also observed in the triple mutant (*SOD1-CuRE1/2/3*_*mut*_) when cells were treated with Cu. Additionally, despite mutating both CuRE1 and CuRE2, the added mutation of CuRE3 in the triple mutant prevented *SOD1* de-repression as seen in the *SOD1-CuRE1/2*_*mut*_ strain during Cu deficiency (Fig. 3*C*). This suggests while CuRE1 and CuRE2 are absolutely required for *SOD1* repression during Cu deficiency, CuRE3 may have a role in activating *SOD1* expression by some unknown mechanism that is independent of Cu status. These changes in transcript levels were reflected at the level of Sod1 protein as assessed by immunoblot for the WT, *SOD1FLAG*, *SOD1*^*C*^, and *SOD1-CuRE1/2*_*mut*_ strains (Fig. 3*D*). These mutants express high levels of Sod1 protein in the presence of Cu. However, cells experiencing Cu deficiency nearly completely extinguish Sod1 expression, with the exception of the CuRE1/2 mutant which can no longer repress the expression of *SOD1* through Cuf1. While transcript levels were decreased in the *SOD1-CuRE3*_*mut*_ and *SOD1-CuRE1/2/3*_*mut*_ strains compared with the *SOD1*^*C*^ strain when treated with Cu, any strain harboring the CuRE3 mutation failed to express sufficient levels of Sod1 protein under either condition tested (Fig. 3*D*). Whether this is a direct result of a requirement for CuRE3 in Cuf1-mediated activation of Sod1 or if mutation of the CuRE3 site perturbed proper splicing of the *SOD1* transcript requires further investigation. However, given the established dampening of *SOD1* expression in *C. albicans* under Cu limitation as a mechanism to preserve available Cu cofactor (51), the remaining studies here focused on the unknown role of Cuf1 in the regulation of *SOD2*.

**Figure 4:**
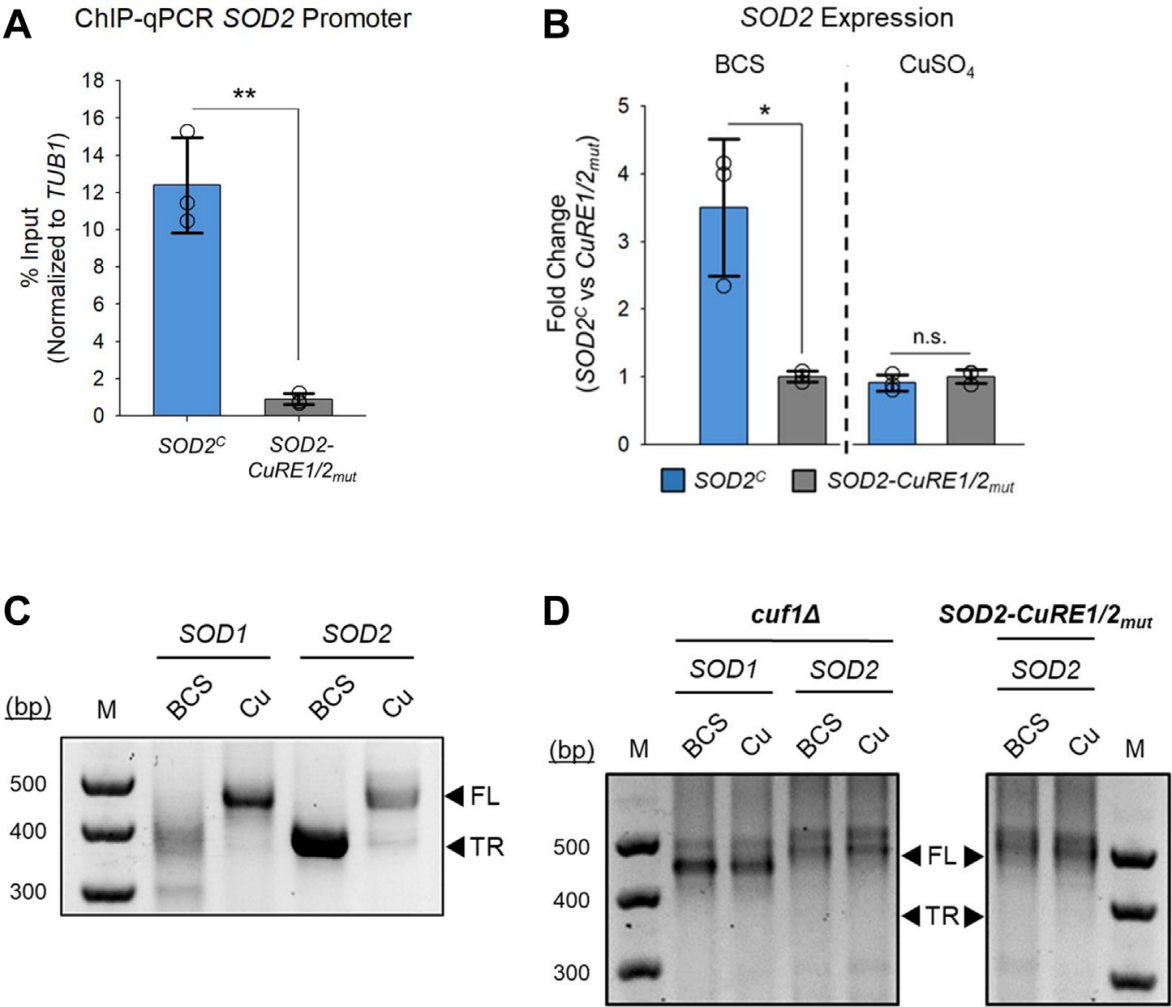
**Cuf1 regulates SOD2 expression through CuRE sequences resulting in an alternative transcript shortened at the 5’-end**. *A*, ChIP-qPCR of the *SOD2* promoter region in the *SOD2*^*C*^ and *SOD2-CuRE1/2*_*mut*_ strains containing the *CUF1*-*FLAG* allele when treated with 1 mM BCS. N = 3, 2-tailed *t* test. *B*, qRT-PCR analysis of *SOD2* transcript levels in the *SOD2*^*C*^ and *SOD2-CuRE1/2*_*mut*_ strains when treated with 1 mM BCS or 0.3 mM CuSO_4_. N = 3, 2-tailed *t* test. *C*, visualization of final 5’-RACE PCR products of *SOD1* and *SOD2* from WT *C. neoformans* cells treated with 1 mM BCS or 5 μM CuSO_4_. Products were separated by agarose gel electrophoresis and visualized by ethidium bromide staining. *D*, Same as in (*C*), but using the *cuf1Δ* strain and the *SOD2-CuRE1/2*_*mut*_ strain. ∗*p* < 0.05, ∗∗*p* < 0.01, n.s., not significant. BCS, bathocuproinedisulfonic acid; CuRE, copper responsive element; FL, full length; TR, truncated, shortened; SOD, superoxide dismutase.

To interrogate roles for the putative CuRE sites within the *SOD2* promoter (Fig. 3*A*), a *sod2Δ* strain was created by replacement of the *SOD2* allele with a nourseothricin (N-acetyltransferase [NAT]) cassette. A WT copy of *SOD2* harboring its native promoter and an additional carboxyl-terminal HA epitope tag (*SOD2*^*C*^), or a *SOD2* allele with CuRE sites mutated individually (*SOD2-CuRE1*_*mut*_ and *SOD2-CuRE2*_*mut*_) or in combination (*SOD2-CuRE1/2*_*mut*_) in the *SOD2* promoter was re-introduced within the safe haven locus (50) of the *sod2Δ* strain. In the same strains, a functional *CUF1* allele at its native locus was generated with a 4x-FLAG epitope tag for ChIP studies. The binding of Cuf1 at the *SOD2* promoter and regulation of *SOD2* from these alleles were assayed by ChIP-qPCR and qRT-PCR, respectively. Cells cultured in the presence of BCS exhibited a strong enrichment of Cuf1 within the *SOD2* promoter region in the *SOD2*^*C*^ strain. However, mutation of consensus nucleotides within the putative CuRE sequences in the *SOD2*-*CuRE1/2*_*mut*_ strain resulted in little enrichment of Cuf1 at the *SOD2* promoter as evidenced by ChIP-PCR (Fig. 4*A*), validating these CuREs as *bona fide* Cuf1 binding sites *in vivo*. Additionally, the *SOD2*-*CuRE1/2*_*mut*_ strain was defective in inducing expression of *SOD2* in Cu limiting conditions as compared with the *SOD2*^*C*^ strain, whereas there was no differential regulation of *SOD2* between the two strains when cells were supplemented with Cu, as evidenced by qRT-PCR (Fig. 4*B*).

### Cuf1 activation of SOD1 and SOD2 generates 5’-truncated transcripts

To characterize the transcripts produced from the *SOD1* and *SOD2* genes in response to changes in Cu availability, mRNA was purified from *C. neoformans* cells cultured in the presence of Cu or BCS and the 5’-ends of the transcripts mapped by 5’-rapid amplification of cDNA ends (5’-RACE). 5’-RACE confirmed the production of shortened transcripts for both *SOD1* and *SOD2* in response to Cu limitation (Fig. 4*C*). Further, as expected, production of the shortened transcripts was dependent on Cuf1, and for *SOD2*, the two CuRE sequences within its promoter were required for this regulation (Fig. 4*D* and Fig. S3*A*). While a discrete shorter transcript for *SOD2* was produced and sequenced, several species of shorter transcripts were produced from the *SOD1* gene under Cu limitation, which proved difficult to precisely map their 5’-ends. However, it is evident that the full-length *SOD1* transcript is no longer detected in Cu-deficient cells (Fig. 4*C*) and consistent with the mRNA-seq data, suggesting the first exon is no longer transcribed. Skipping of exon 1, which is only nine nucleotides long and encodes the translation initiating methionine codon (ATG), would result in a shift of the reading frame and the insertion of several premature stop codons throughout the truncated *SOD1* transcript (Fig. S3*B*). Interestingly, the 5’-start site for the *SOD2* transcript mapped to the −3 nt position in BCS-treated cells, which maintains the coding potential for a full-length Sod2 protein retaining the mitochondrial import sequence (Fig. S3*B*). However, studies in *S. cerevisiae* and other eukaryotes demonstrate that leaderless or short-leader transcripts are inefficiently translated at the first initiation codon and as a result of leaky ribosome scanning, preferentially begin translation at a downstream initiation codon (52, 53). Examination of the full *SOD2* open reading frame revealed a second putative translation initiation codon in exon 1 beginning at +64 nt which would maintain the native reading frame but result in an amino-terminally truncated polypeptide starting at Met22 (Fig. S3*B*). Using the SignalP signal peptide predictor software (54), this methionine residue immediately precedes the predicted Sod2 mitochondrial import peptide cleavage site between Ala27 and Lys28. Functionally, translation initiation at Met22 would preclude the synthesis of the amino-terminal mitochondrial import peptide and potentially allow for cytosolic accumulation of Sod2.

### The truncated SOD2 transcript encodes two isoforms of Sod2

**Figure 5:**
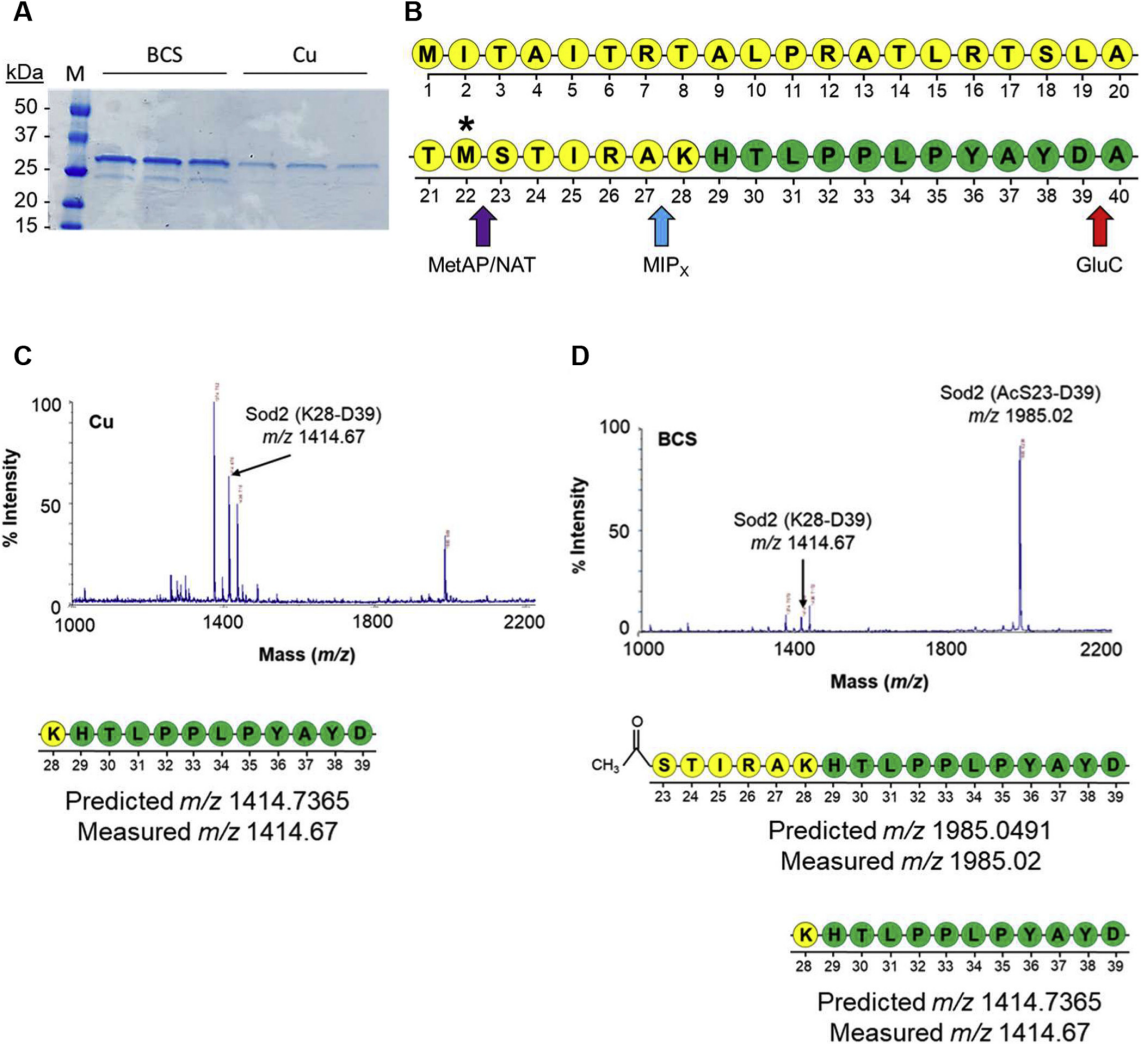
***C. neoformans* expresses an amino-terminally truncated Sod2 protein during Cu limitation**. *A*, SDS-PAGE and Coomassie stain visualization of immunopurified Sod2-HA from cells supplemented with 1 mM BCS or 5 μM CuSO_4_. *B*, schematic representation of the amino-terminus of full length Sod2 protein. Residues in *yellow* represent the mitochondrial import sequence (MIP). *Asterisk* represents location of alternative translation initiation at Met22 from shortened transcript. *Arrows* represent peptide cleavage sites of methionine aminopeptidase (MetAP) and N-acetyl transferase (NAT) (*purple*), MIP cleavage (MIPx) (*blue*), and GluC proteolysis for MALDI-MS (*red*). *C*, MALDI-MS spectrum of GluC digested Sod2-HA peptide fragments purified from cells cultured with 5 μM CuSO_4_. Corresponding schematic of peptide fragment with predicted and observed mass-to-charge (*m/z*) ratios are indicated below. *D*, MALDI-MS spectrum as in (*C*), but from cells cultured with 1 mM BCS. BCS, bathocuproinedisulfonic acid.

To understand the functional implications for generating a truncated *SOD2* transcript under Cu limiting growth conditions, Sod2-HA protein was immunopurified from soluble cell extracts of *C. neoformans* cells cultured in the presence of Cu or BCS (Fig. 5*A*) and purified Sod2-HA protein analyzed by Edman degradation to sequence the amino terminus of the protein. Surprisingly, under both conditions, only a Sod2 protein was detected with the amino terminus beginning at Lys28 (Table S2). While this is significantly downstream of Met1, upon protein translocation into the mitochondria, a peptidase cleaves the mitochondrial import sequence, followed by protein folding around its Mn metal ion to yield the mature Sod2 protein. Based on the *C. neoformans* Sod2 primary sequence, this cleavage site is predicted to be between amino acids Ala27 and Lys28 and correlates with the Edman sequencing results (Fig. 5*B*). However, this sequencing reaction is dependent upon the presence of a free amine group at the amino terminus, and in yeast, cytosolic proteins are commonly posttranslationally modified by methionine aminopeptidases to cleave the amino-terminal methionine, followed by acetylation of the amino-terminal primary amine group by NATs (55, 56). Acetylation of the amino terminus of a cytosolic isoform of Sod2 would inhibit the degradation reaction and preclude detection by Edman sequencing (Fig. 5*B*).

To test this hypothesis, purified Sod2-HA was proteolytically digested with the endopeptidase GluC, peptide fragments extracted, and analyzed by MALDI-MS/MS. As expected, Sod2 from cells grown in the presence of Cu revealed amino-terminal fragments corresponding to peptides beginning at Lys28 (*m/z* 1414.67) (Fig. 5*C*). However, Sod2 from cells grown under Cu-deficient conditions clearly revealed amino-terminal fragments corresponding to a Sod2 protein that initiated at Met22. The mass of this peptide (*m/z* 1985.02) correlated to that of a Sod2 peptide that initiated translation at Met22 was subsequently amino-terminally cleaved by a methionine aminopeptidase and further modified with an acetyl group at its amino terminus (Fig. 5*D*), confirming the generation of a new Sod2 isoform lacking the mitochondrial import sequence under Cu limiting growth conditions. Importantly, the full-length mitochondrial isoform (*m/z* 1414.67) was also readily detected from cells grown under Cu-limiting conditions (Fig. 5*D*).

### Translation initiation at Met22 results in cytosolic accumulation of Sod2

**Figure 6:**
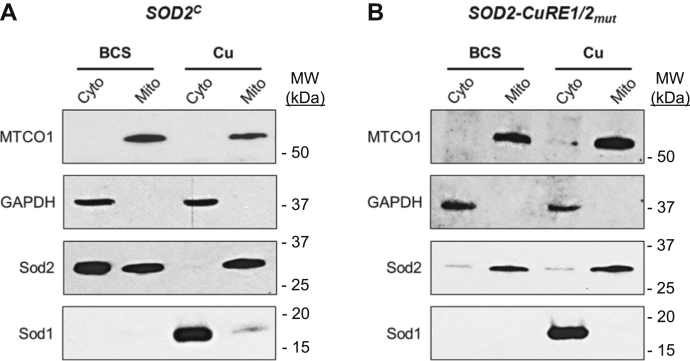
**Truncated Sod2 protein accumulates in the cytosol**. *A*, *SOD2*^*C*^ cells supplemented with 1 mM BCS or 5 μM CuSO_4_ were assayed for Sod1 and Sod2 protein localization after subcellular fractionation of crude mitochondrial extracts from the cytosolic fraction and visualized by Western blotting. Control proteins specific to cytosol (GAPDH—glyceraldehyde-3-phosphate dehydrogenase) and mitochondria (MTCO1—mitochondrial COX1 subunit) were included to validate fractionation efficacy. *B*, Subcellular fractionation as in (*A*) but using the *SOD2-CuRE1/2*_*mut*_ strain. BCS, bathocuproinedisulfonic acid.

To evaluate the localization of the Sod2 proteins expressed from the 5’-truncated transcript, subcellular fractionation was conducted to isolate crude mitochondrial extracts and postmitochondrial supernatants (cytosol). As expected, Cu-sufficient cells expressed predominantly mitochondrially localized Sod2 (Fig. 6*A*). However, cells grown under Cu-limiting conditions demonstrated significant accumulation of Sod2 in the cytosol, with levels of mitochondrial Sod2 approximately equivalent to that found in cells grown under Cu-replete conditions. High levels of cytosolic Sod1 were detected during Cu sufficiency, with a small fraction associated with the mitochondria, presumably Sod1 that localizes to the mitochondrial IMS. However, under Cu-deficiency conditions, *C. neoformans* exhibited near complete loss of Sod1 protein. Thus, when cells are Cu deficient, Cuf1 represses expression of the highly abundant Cu-dependent protein Sod1 and directs the expression of a cytosolic isoform of Mn-dependent Sod2. In contrast, mutation of the *SOD2* CuRE sites, which abolishes Cuf1 binding and Cuf1-dependent activation of the shorter *SOD2* transcript, fails to drive expression of the cytosolic isoform of Sod2 during Cu limitation, and Sod2 is primarily targeted to the mitochondria (Fig. 6*B*). However, a low level of cytosolic Sod2 was detected from these fractionation assays, which could be because of a small fraction of lysed mitochondria or newly synthesized Sod2 peptide that has yet to translocate to the mitochondria. Regardless, mutation of the CuRE sites clearly results in the failure of Cuf1 to drive cytosolic localization of Sod2.

### Cytosolic Sod2 protects *C. neoformans* from oxidative stress during Cu limitation

**Figure 7:**
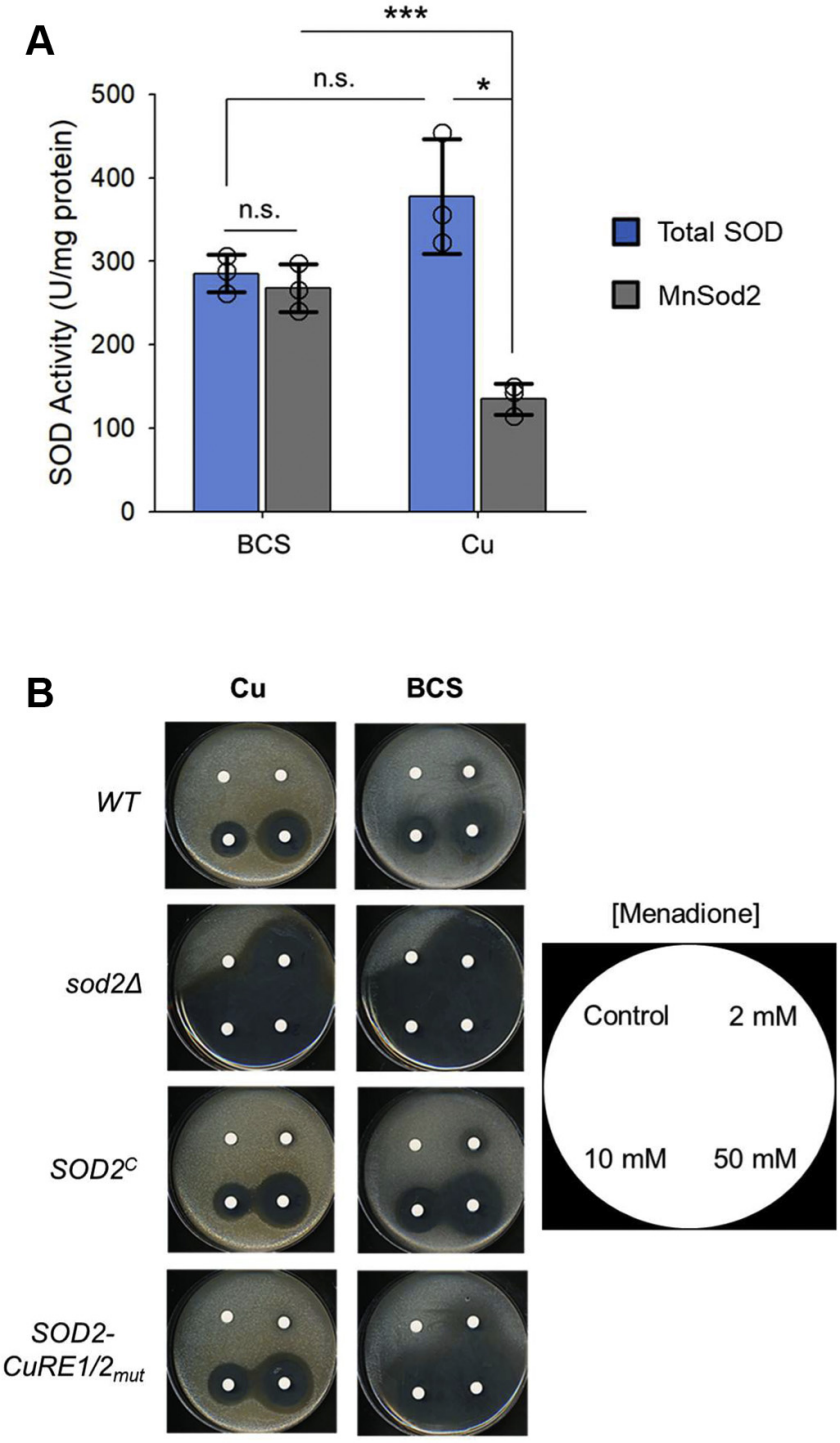
**Truncated Sod2 is protective against superoxide stress during Cu limitation**. *A*, *in vitro* total SOD activity assays of whole cell extracts from WT *C. neoformans* cells supplemented with 1 mM BCS or 0.1 mM CuSO_4_. Assays were repeated in the presence of KCN to detect SOD activity because of the contribution of Sod2. N = 3, 2-tailed *t* test. *B*, *In vivo* cellular superoxide sensitivities as tested by disc diffusion assays in the indicated strains and in the presence of 1 mM BCS or 0.1 mM CuSO_4_. Cells were challenged by spotting increasing concentrations of the superoxide generator menadione on filter discs and allowing cells to incubate at 30 °C for 3 days. Menadione concentrations spotted onto discs are listed on key to the right. ∗*p* < 0.05, ∗∗∗*p* < 0.005, n.s., not significant. BCS, bathocuproinedisulfonic acid; SOD, superoxide dismutase.

To test whether the cytosolic isoform of Sod2 is capable of buffering the cytosolic compartment from superoxide stress, SOD activity was measured from soluble cellular extracts. There was a slight but nonstatistically significant reduction in total SOD activity between WT cells grown under Cu-deficient *versus* Cu-supplemented growth conditions, indicating overall superoxide buffering capacity within *C. neoformans* is relatively unperturbed (Fig. 7*A*). These assays were repeated in the presence of KCN, which specifically inhibits Cu, Zn-Sod1 activity, revealing the SOD activity contributed by Sod2, and by subtraction, infers the activity attributed to Sod1. In cells treated with Cu, Sod2 accounts for approximately 30% of the total SOD activity. Corroborating the findings that Sod1 is repressed during Cu limitation, nearly 100% of the total SOD activity is due to Sod2 under Cu-limiting conditions (Fig. 7*A*). These results demonstrate that the Cuf1-dependent repression of Sod1 and induction of both the cytosolic and mitochondrial isoforms of Sod2 during Cu limitation maintain normal overall superoxide buffering capacity in *C. neoformans*.

To ascertain whether the reallocation of SOD activity in the cytosol is sufficient to protect *C. neoformans* from superoxide stress *in vivo*, isogenic WT, *sod2Δ*, *SOD2*^*C*^, and *SOD2-CuRE1/2*_*mut*_ strains were grown in the presence of the superoxide generator menadione under either Cu-sufficient or Cu-limiting conditions. Filter discs soaked in increasing concentrations of menadione were used to challenge *C. neoformans* growth by disc diffusion assays (Fig. 7*B*). Consistent with the *in vitro* SOD activity assays, there was increased sensitivity as evidenced by a slightly larger zone of growth inhibition of WT cells treated with BCS compared with Cu, indicating that cytosolic Sod2 is able to protect *C. neoformans* from oxidative stress during Cu limitation. However, the *sod2Δ* strain was sensitive to menadione under all conditions tested, as the complete loss of mitochondrial Sod2 results in severe oxidative damage within the mitochondria. Menadione resistance was restored to WT levels in the *SOD2*^*C*^ complemented strain. While the *SOD2*-*CuRE1/2*_*mut*_ strain demonstrated WT levels of menadione resistance when supplemented with Cu, this strain exhibited severe sensitivity to menadione when grown in the presence of BCS. This reactive oxygen stress-sensitivity was specific to superoxide, as treatment with hydrogen peroxide did not show any differential reactive oxygen stress protection between any of the isogenic strains (Fig. S4). Thus, failure of Cuf1 to bind to the CuRE sequences within the *SOD2* promoter activates expression of the shortened *SOD2* transcript and in turn generates the cytosolic isoform of Sod2, leading to oxidative stress sensitivity during Cu-limitation, a condition when Cu, Zn *SOD1* expression is simultaneously inhibited by Cuf1.

### Cytosolic Sod2 impacts colonization of the brain during cryptococcal meningitis

SODs are well-established virulence determinants in both prokaryotic and eukaryotic pathogens. This is because of their role in resisting the host-imposed oxidative burst within phagocytic immune cells and in other infectious compartments. The normal infectious cycle of *C. neoformans* begins after inhalation of desiccated spores or yeast into the pulmonary tract where they colonize the lung. Live animal imaging demonstrates that *C. neoformans* senses a high Cu environment within this host niche, and the Cu detoxification response (*MT1/2*), directed by Cuf1, is critical in mounting a defense and for successful colonization (26). However, as infection persists and progresses, *C. neoformans* disseminates from the lungs to the bloodstream and crosses the blood–brain barrier, resulting in colonization and often fatal meningitis. Within the brain, *C. neoformans* senses Cu-limitation, and activation of the Cu-acquisition machinery (*CTR1/4*) by Cuf1 is critical for brain colonization (25). Additionally, transcriptome studies of a *C. neoformans* clinical isolate grown in human cerebral spinal fluid demonstrated the Cu-deficiency response was activated (57). However, a different clinical isolate displayed the opposite response, with the Cu detoxification response activated, thus lending some uncertainty as to the actual Cu status in macrophages *in vitro* (5, 57). Given the reduction of *SOD1* expression, Cuf1 regulation of *SOD2* may be critical for defense against host-derived ROS in Cu-deficient infectious niches.

**Figure 8:**
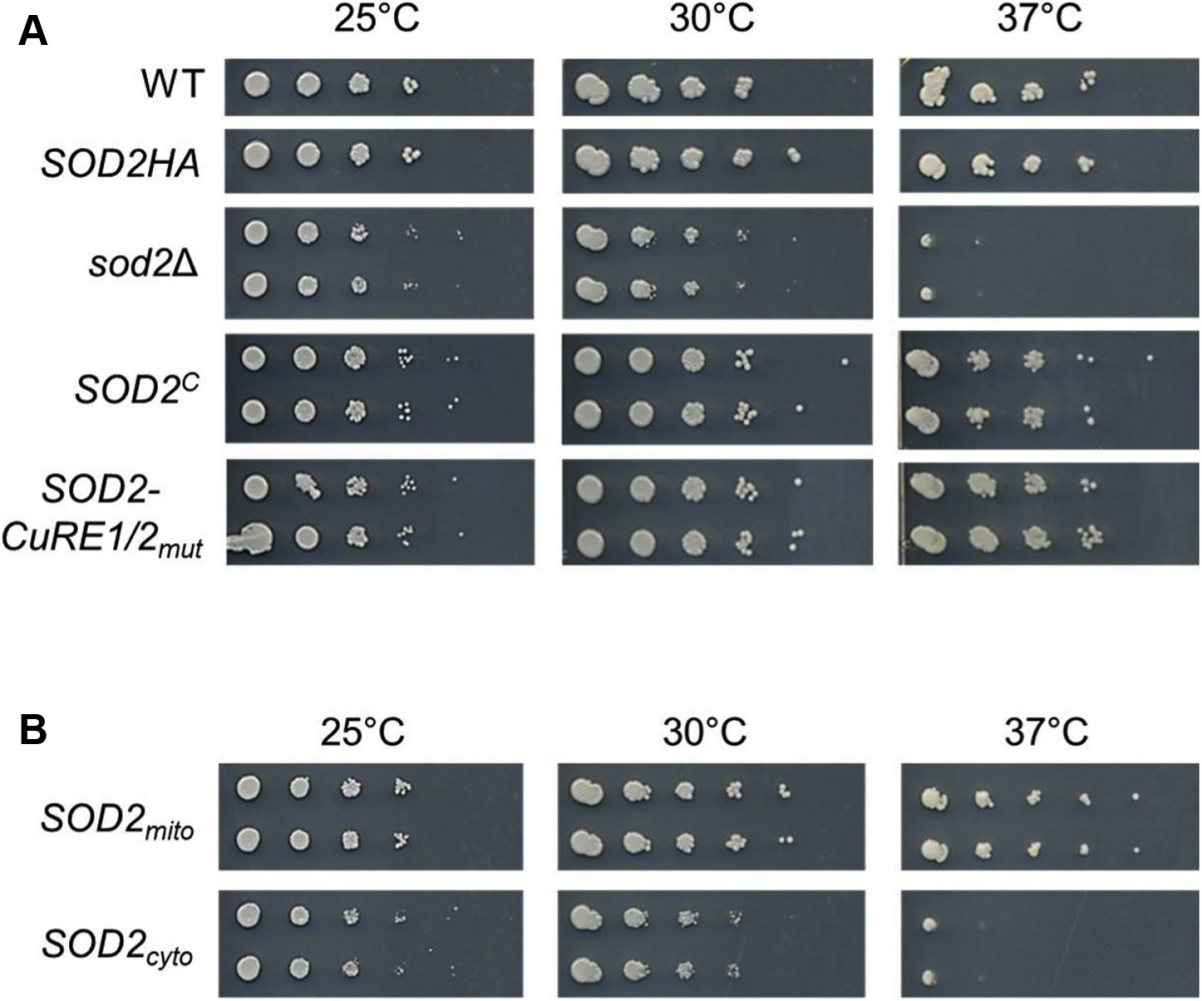
**Mitochondrial localized Sod2 is required for growth at elevated temperatures.***A*, The indicated strains were serially diluted and spotted onto SC agar plates, incubated at 25 °C, 30 °C, or 37 °C for 3 to 4 days and photographed. *B*, Same as in (*A*), but using strains only expressing mitochondrial Sod2 (*SOD2*_*mito*_) or only expressing cytosolic Sod2 (*SOD2*_*cyto*_). SC, synthetic complete; SOD, superoxide dismutase.

Previous work demonstrated that *C. neoformans sod2Δ* cells are avirulent in murine models of cryptococcosis (58). One of the most striking phenotypes of *sod2Δ* mutants is severe temperature sensitivity at 37 °C (Fig. 8*A*). This likely contributes strongly to the avirulent phenotype in murine models of infection. Both the *SOD2*^*C*^ and *SOD2-CuRE1/2*_*mut*_ strains complemented the temperature-sensitive phenotype exhibited by the *sod2Δ* allele in a colony spotting assay on synthetic complete (SC) agar plates (Fig. 8*A*). To further test the role of Sod2 in thermoprotection, Sod2-expressing strains were generated in which the genomic *SOD2* DNA, starting from the first initiation codon (Met1, designated *SOD2*_*mito*_) or the second downstream initiation codon (Met22, designated *SOD2*_*cyto*_) was placed under control of the constitutively active actin (*ACT1*) promoter, thereby decoupling Cuf1-dependent activation and the generation of a 5’-shortened *SOD2* transcript. Subcellular fractionation of the *SOD2*_*mito*_ and *SOD2*_*cyto*_ strains grown under either Cu-limiting or Cu-sufficient conditions confirmed the solely mitochondria or cytosolic localization of Sod2, respectively, regardless of Cu status (Fig. S5, *A* and *B*). Expression of the *SOD2*_*mito*_ allele restored growth at high temperatures. However, expression of *SOD2*_*cyto*_ remained temperature sensitive, indicating that mitochondrial localized Sod2 is required for growth at elevated temperatures (Fig. 8*B*).

**Figure 9:**
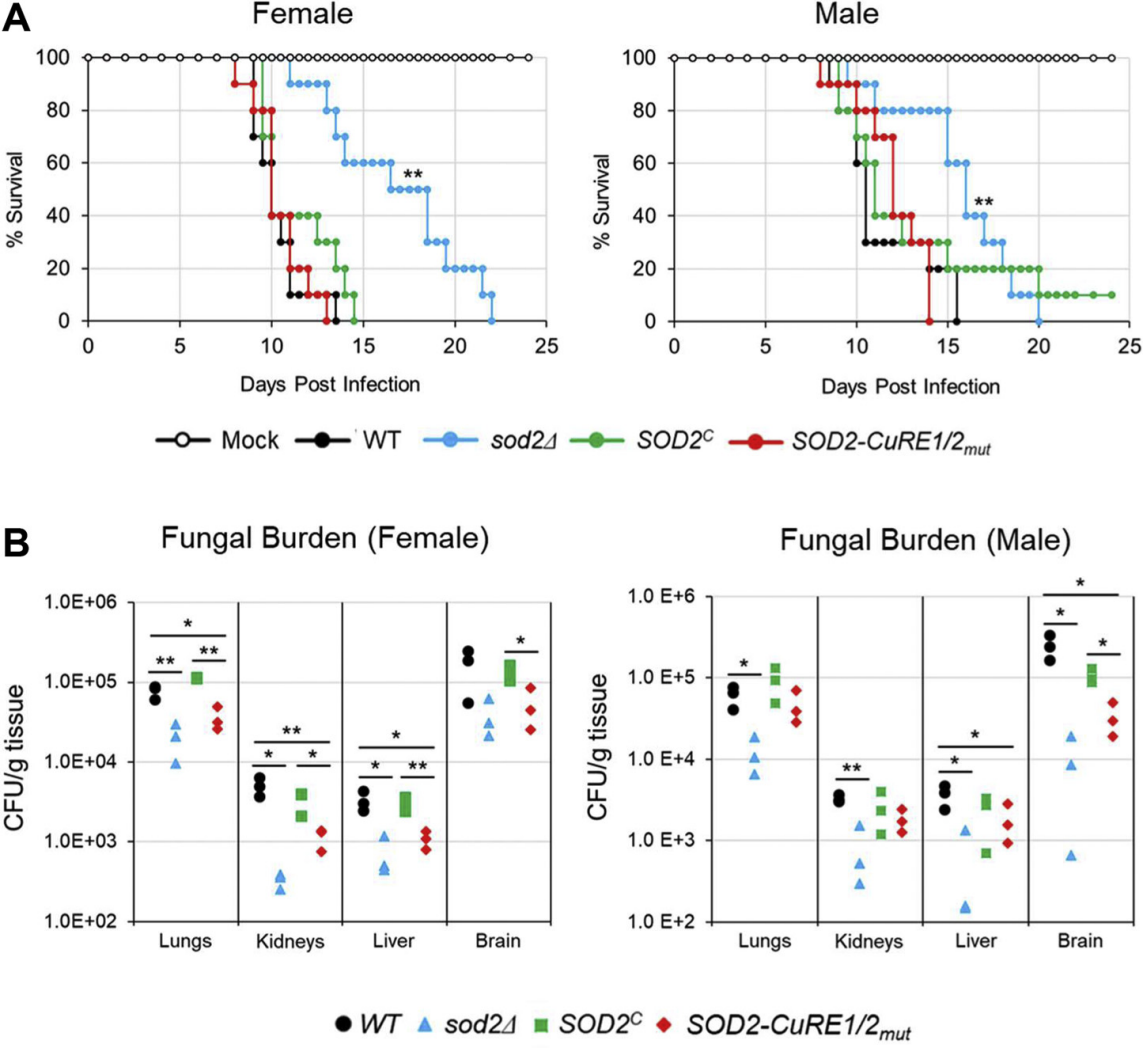
**Cytosolic Sod2 is required for colonization of Cu-deficient host niches using murine models of fungal infection**. *A*, retro-orbital administration of 500 cells of the indicated strains resuspended in 1X PBS into A/J male and female mice. Shown are mouse survival plots with percent survival plotted as a function of days postinfection. Strain genotypes are indicated, and mock infected mice were administered an equal volume of 1X PBS. The survival average of mice infected with the wildtype (WT) was compared with the survival average of mice infected with any of the mutants by the log rank statistical test. N = 10. *B*, *C. neoformans* CFU analysis of mouse organs 3 days postinfection. Shown are the indicated *C. neoformans* genotypes. Statistical differences were assessed across groups by the nonparametric Kruskal-Wallis one-way analysis of variance. F-test for variances was used between sample groups and significance determined by a 2-tailed t-test. N = 3. ∗*p* < 0.05, ∗∗*p* < 0.01, ∗∗∗*p* < 0.005. CFU, colony forming unit.

To evaluate the potential role of Sod2 localization in tissue colonization and *C. neoformans* virulence, studies were conducted in murine models of infection by retro-orbital administration in both male and female A/J mice. This administration route bypasses the high Cu environment of the lung, resulting in direct bloodstream dissemination of *C. neoformans* providing access to various organs and tissues, and ultimately to the brain. Mice infected with WT cells succumb rapidly by day 11 (Fig. 9*A*). Infection with the *sod2Δ* strain resulted in a hypovirulent phenotype, with mouse survival significantly prolonged. This result was surprising, given previous reports of strains carrying the *sod2Δ* allele being avirulent, and data shown herein demonstrating that the *sod2Δ* strain is unable to grow *in vitro* at 37 °C (see Discussion) (58). As expected, the *SOD2*^*C*^ strain restored full virulence to the *sod2Δ* strain. However, the *SOD2-CuRE1/2*_*mut*_ strain showed a similar trend to that of WT cells, displaying no clear difference in virulence (Fig. 9*A*).

In addition to the brain, retro-orbital administration allows access of the pathogen to other host compartments, perhaps allowing *C. neoformans* to colonize alternative Cu sufficient niches. To assess this, colony forming units (CFU)s in several organs were quantitated. At 3 days postinfection, both the *sod2Δ* and *SOD2-CuRE1/2*_*mut*_ strains showed reductions in CFUs in all organs tested when compared to the WT and *SOD2*^*C*^ strains (Fig. 9*B*). At 7 days postinfection, the CFUs from infection with the *sod2Δ* strain remained significantly lower in all tissues tested, except the lung, whereas the *SOD2-CuRE1/2*_*mut*_ mutant reached colonization levels similar to that of the WT and *SOD2*^*C*^ strain (Fig. S6). These results suggest that while the host immune system contains the infection at late times after infection, the *SOD2-CuRE1/2*_*mut*_ strain has a disadvantage in tissue colonization at early stages of infection, particularly in the Cu-limited growth environment of the brain. We observed no significant differences in mouse survival nor organ CFUs when comparing male *versus* female mice.

## Discussion

DNA binding transcription factors have the capacity to re-program cellular networks to cope with fluctuations in environmental conditions. With this in view, Cuf1 is multifunctional in copper biology in that it drives adaptive responses to Cu status in *C. neoformans* through several mechanisms. First, under Cu-limiting growth conditions, Cuf1 activates the transcription of genes such as those involved in Cu^+^ import, and other functions, that allow cells to adapt to low levels of this essential trace element (25, 42, 43). Second, upon exposure to high Cu levels, Cuf1 drives the expression of genes that function in Cu detoxification, such as MTs and the Atm1 FeS biogenesis protein, that allow cells to detoxify and adapt to potentially toxic Cu levels (26, 42, 59). Of all the Cu metalloregulatory transcription factors characterized from algae to fungi to mammals, Cuf1 has the unusual capacity to respond to both Cu limitation and Cu excess through driving changes in transcript abundance.

Here, we identify a third level of cellular adaptation to changes in Cu availability by Cuf1, *via* the profound redistribution of protein subcellular localization. In this process, *C. neoformans* not only reciprocally regulates expression of the SODs, *SOD1* and *SOD2*, during Cu deficiency but also appears to activate the expression of *SOD1* during Cu sufficiency. While Cuf1 was seen to be enriched at the promoter of *SOD1* under both Cu sufficient and Cu deficient conditions, the recognition sequence, mechanism of activation, and the role of CuRE3 in *SOD1* regulation during Cu sufficiency remains to be determined. In contrast, during Cu deficiency Cuf1 drives the expression of alternative transcripts by specifically recognizing and binding to CuRE sequences within their promoter regions to generate 5’-shortened transcripts. The CuRE sequences are located downstream of the normal transcription initiation sites, thereby likely Cuf1 influences RNA Pol II site selection for transcription initiation or influences other RNA processing machinery. This results in the repression of *SOD1* expression through the generation of a transcript with multiple translation termination codons. Moreover, Cuf1 binds to the *SOD2* promoter, generating a shorter transcript that switches expression from a Sod2 protein solely localized to the mitochondrial lumen to the expression of two Sod2 isoforms from a single truncated RNA transcript: the canonical mitochondrial targeted isoform and a new cytosolic isoform lacking the amino-terminal encoded MIP. In this new environment, the cytosolic isoform of Sod2 maintains activity and is required for protection of cells against superoxide stress. Additionally, induction of the cytosolic isoform of *SOD2* contributes to the ability of *C. neoformans* to colonize specific niches within the host during infection. This was particularly evident during cryptococcal colonization of the brain and may impact the pathogenic potential and ability of this organism to colonize Cu deficient host tissues during acute stages of infection. As *C. neoformans* predominantly impacts immunocompromised individuals, the requirement for the cytosolic relocalization of Sod2 may have particular importance when innate immune cells are unable to efficiently clear the invading pathogen.

In eukaryotes, the ribosome preinitiation complex, along with other initiating factors, recognizes the 5’-cap of mRNA transcripts and scans downstream until the initiating AUG codon is recognized. In this ribosome scanning model in *C. neoformans*, start codon selection is dictated by the context of the surrounding Kozac consensus sequence, as well as the proximity of the start codon to the 5’-cap (60). Start codons with a weak Kozac context or that are near the 5’-cap of mRNA transcripts (<15 nt) are prone to leaky ribosome scanning, where there is an inherent inefficiency at recognizing and initiating translation at the first AUG codon, and instead initiates at a downstream AUG codon (60, 61). For *SOD2*, Cuf1-dependent regulation activates expression of a transcript with an extremely short (3 nt) leader sequence which may be prone to leaky ribosome scanning, with translation initiating at a downstream start codon. The first downstream AUG codon occurs immediately preceding the encoded MIP, allowing for the generation of a new cytosolic isoform of Sod2. This mechanism of dual localization of mitochondrial targeted proteins appears to be a common theme among transcripts prone to leaky ribosome scanning. For example, amino-acyl tRNA synthetases are required for cytosolic as well as mitochondrial localization for the synthesis of proteins within these respective compartments, and many organisms encode dedicated genes for cytosolic or mitochondrial isoforms (62, 63). However, *C. neoformans* typically encodes only a single copy of the amino-acyl tRNA synthetases genes and regulates their localization by leaky ribosome scanning, in which two isoforms are produced from the same mRNA transcript, perhaps in an analogous manner to what we have discovered for Sod2 (60).

Interestingly, despite producing only a truncated transcript under Cu-limiting conditions, our results demonstrate that WT levels of mitochondrial Sod2 levels are maintained. Three hypothesis could explain this: (1) a small but significant fraction of full length *SOD2* mRNA transcript is generated to produce the mitochondrially targeted Sod2 protein; (2) the loss in efficiency of initiation at the canonical start codon is overcome by inducing the level of *SOD2* transcript produced, or (3) additional initiation factors are involved in guiding the preinitiation complex to recognize the canonical start codon, in combination with leaky ribosome scanning, to produce both isoforms of Sod2. Our data argue against the first hypothesis, as we were unable to detect a full-length transcript by 5’-RACE; however, this possibility cannot be ruled out by these experiments alone. While additional regulatory factors may be activated during Cu deficiency that guide ribosomes to the downstream initiation codon of the *SOD2* transcript, the fact we see no induction of the cytosolic isoform of Sod2 in the *SOD2-CuRE1/2*_*mut*_ strain would argue against this hypothesis. However, the shortened transcript may be required for specific interactions with these hypothetical regulatory factors. Thus, we currently favor the second hypothesis in which both isoforms are translated by a leaky ribosome scanning mechanism. However, additional studies are needed to test these models.

In the face of Cu limitation, cytosolic Sod2 supports cellular superoxide detoxification in the absence of Sod1. This end result is analogous to what has been previously demonstrated in *C. albicans*, in which a separately encoded Mn-dependent *SOD3* gene is induced during Cu limitation to replace cytosolic Sod1 (24, 51). This regulation is mediated by the low Cu-sensing transcription factor Mac1 which recognizes CuRE sequences in the promoter of *SOD3* and within an intronic region of *SOD1* to activate and repress their expression, respectively (24). However, *C. neoformans* does not encode a *SOD3* gene and instead has evolved a different strategy to drive the simultaneous dual localization of the normally mitochondrial targeted Sod2. Also unique to *C. neoformans*, two CuRE sequences are present within the promoters of *SOD1* and *SOD2* which are required for their repression and activation, respectively, during Cu limitation. It is still unclear as to the function (if any) of the third CuRE site within the first intron of *SOD1*. While this functions as a protective measure against reactive oxygen stress in the absence of sufficient Cu for Sod1, the additional repression of Sod1 may serve as a Cu sparing mechanism by reducing the cellular Cu quota or re-prioritizing this essential trace element in times of Cu limitation. This “metal sparing” is an efficient mechanism that allow cells to adapt to metal-limiting environments by repressing highly abundant metal dependent enzymes and replacing them with alternative cofactor requiring enzymes performing similar or analogous functions (64, 65). One of the most elegant examples demonstrating this is in the photosynthetic algae *Chlamydomnas reinhardtii*. When faced with Cu limitation, the highly abundant Cu protein plastocyanin is repressed and is replaced by the induction of an alternative heme-dependent protein cytochrome c6 (66). Additionally, preexisting pools of plastocyanin are targeted for degradation, and its Cu cofactor recycled to support more essential cellular processes, such as to multicopper oxidases for high affinity iron uptake or to cytochrome c oxidase for cellular respiration (66). An analogous function may occur in *C. neoformans*, in which the ubiquitous Sod1 protein is repressed to lower the cellular demand for Cu. Support of this mechanism in pathogenic fungi has recently been established in *C. albicans*, where a *mac1*Δ/Δ mutant unable to repress *SOD1* expression during Cu deficiency also has a mitochondrial respiratory defect, which is alleviated by introducing the *sod1*Δ/Δ mutation (67).

While cytosolic Sod2 is protective against superoxide stress in the absence of cytosolic Sod1, it is curious how superoxide levels within the IMS are buffered. Unfortunately, we were unable to identify a suitable *C. neoformans* control marker to conduct submitochondrial fractionation to delineate IMS and matrix fractions. Thus, we cannot determine the submitochondrial location of Sod2. *C. albicans* reduces superoxide levels within the IMS by inducing the alternative oxidase, *AOX2*, through the Mac1 low Cu-sensing transcription factor (51). AOX2 bypasses complex III in the electron transport chain, one of the major generators of superoxide in the IMS, thus maintaining low IMS levels of superoxide in the absence of Sod1. We were unsuccessful in identifying a putative *C. neoformans AOX2* by bioinformatic analysis using *C. albicans AOX2* as a template. While *C. neoformans* does harbor a gene for *AOX1*, we saw no increase in the abundance of its transcript during Cu deficiency (Fig. S7), suggesting that there is no transcriptional regulation of *AOX1* during Cu limitation. Further experimentation is required to determine the mechanism by which *C. neoformans* maintains superoxide levels in the IMS during Cu deficiency.

The localization of cytosolic Sod2 raises the interesting question of how Mn is delivered to Sod2 within this new cellular compartment? It is not well understood how the Sod2 protein is metallated in the mitochondria, but reports from studies in *S. cerevisiae* suggest that Mn must be routed through the Smf2 Mn transporter, located within intracellular vesicles, for proper activation of Sod2 (45). Additionally, a potential mitochondrial inner membrane chaperone protein, Mtm1, is implicated in the proper insertion of Mn into Sod2 while it is translocated into the mitochondrial lumen (41). Interestingly, inhibition of Sod2 translocation to mitochondria or expression of a truncated isoform of Sod2 lacking the MIP in *S. cerevisiae*, both of which result in cytosolic accumulation of Sod2, demonstrate Sod2 is folded within the cytoplasm but has no activity because of the inability to acquire the Mn metal cofactor (68). Only by culturing cells in high concentrations of Mn (>100 μM) was partial SOD activity restored, suggesting that Mn insertion into Sod2 occurs in a semifolded state within the mitochondria (68). How Mn is inserted into Sod3 in *C. albicans* or cytosolic Sod2 in *C. neoformans* remains an outstanding question but may require specific cytosolic Mn chaperones or protein folding chaperones to maintain cytosolic Sod2/3 in an unfolded state until activated by Mn. Given that intracellular metal concentrations are tightly regulated to limit the accumulation of free metals, it would seem prudent for cells to have evolved a Mn chaperone to fulfill this function as opposed to acquiring Mn from an intracellular “labile” metal pool.

The switch from cytosolic Cu/Zn-Sod1 to cytosolic Mn-Sod2 during Cu limitation in *C. neoformans* also raises the question of how cells overcome host nutritional immunity mechanisms to acquire sufficient Mn? The innate immune system restricts availability of metals from invading pathogens to starve them of these essential elements. Mn is actively effluxed from pathogen-containing phagosomes within macrophages and neutrophils by the NRAMP1 metal transporter (69, 70, 71, 72). Additionally, neutrophils secrete calprotectin, which can bind and sequester Mn, among other metals, with extremely high affinity, further restricting Mn to the pathogen (73, 74, 75, 76). This would indicate a dynamic interplay between metal availabilities within specific host niches to balance appropriate SOD and other activities that are dependent upon metal status. We tested for any transcriptional activation of known and putative Mn transport genes from cells grown under Cu-limiting or Cu-sufficient conditions to assess if there was a greater demand for cellular Mn accumulation during Cu limitation to sustain cytosolic Sod2. We saw no increased transcript abundance for known and putative *C. neoformans* Mn transporter genes in response to Cu limitation (Fig. S8*A*). However, we also analyzed the total Mn content of cells by inductively coupled plasma mass spectroscopy (ICP-MS) analysis and discovered there was a roughly 30% increase in Mn content of Cu-deficient cells compared with cells that were Cu sufficient (Fig. S8*B*). This would suggest while there is no transcriptional activation of Mn transport genes, there may be increased Mn transport activity in Cu starved cells. Another possibility is there is some other unknown Mn transporter activated under Cu-deficient conditions. Regardless, this raises the interesting possibility of a link between Cu and Mn sensing and homeostasis in these organisms, and activation of a Mn-dependent SOD may be a pathogen-specific adaptation for coping with limited Cu bioavailability within a host.

Finally, while the murine infection models suggest Cuf1 regulation of *SOD2* does not affect the virulence of *C. neoformans*, this regulation does appear to impact the ability of *C. neoformans* to colonize Cu deficient niches, including the brain and kidneys, during acute stages of infection. It was surprising to also observe reduced colonization in the lung, a niche that has previously been described as a high Cu environment, presumably within the phagosome of macrophages (9, 10, 11, 12, 13). Perhaps bypassing the normal inhalation route activates a different response, and host immune cells activate a Cu withholding response as was previously described (27, 28). Also surprising, our results indicate the *sod2Δ* cell line has attenuated virulence, in contrast to being avirulent as previously reported (58). This is counterintuitive considering the temperature-sensitive growth phenotype of this mutant on agar medium and suggests additional antioxidant functions may be activated in the mitochondria during infection conditions that allow for *C. neoformans* to overcome this oxidative stress. Initially, we considered that our *sod2Δ* strain mutated or evolved within the host to adapt to the higher temperature environment of the host. However, these hypotheses were ruled out by spot plating the initial inoculum as well as colonies isolated from different tissues harvested from mice and subjecting them to growth at 37 °C. Both initial *sod2Δ* inocula, as well as cells isolated *in situ* from host tissues, still demonstrated a temperature sensitive phenotype on agar plates (Fig. S9, *A* and *B*). This would imply there may be other compensatory systems activated specifically during infection to overcome the temperature sensitive phenotype demonstrated *in vitro*. Alternatively, it has been demonstrated that the standard housing conditions for mice are at subthermoneutral levels, which imposes a baseline state of chronic moderate stress within mice (77). While core body temperatures remain centered at 37 °C, this chronic moderate stress can modulate the immune response.

While we cannot reconcile these discrepancies between *sod2Δ* phenotypes in virulence, there are some key differences between these two studies. The previous study used C57/BL6 mice, whereas our current study used A/J mice. The field has begun to move away from the C57/BL6 mice when studying the role of metals in nutritional immunity, as it was discovered the C57/BL6 mice contain a mutation in the NRAMP1 transporter, rendering immune cells impaired in classical nutritional immunity responses (78, 79). The previous reports also infected their mice through the traditional respiratory route of infection. We did test the impact of our *sod2Δ* mutant by this infection route as well, yet still this strain displayed attenuated virulence (Fig. S9*C*). Finally, it was discovered after years of circulating the original *C. neoformans* H99 clinically isolated strain between various labs, a lab evolved “wimp” strain emerged that was significantly attenuated in virulence compared to the original “stud” strain (80). While the current studies presented here used the fully virulent “stud” strain, it is uncertain if these discrepancies could be because of the use of the less virulent “wimp” strain in previous studies.

**Figure 10:**
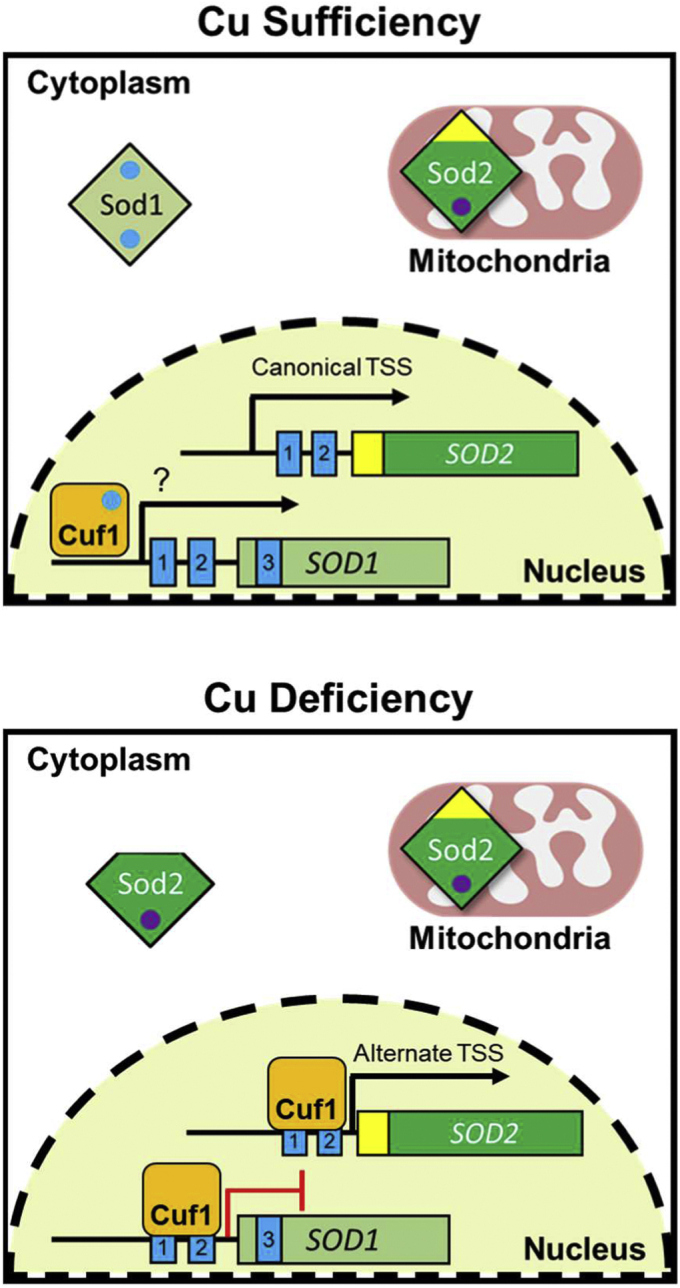
**Model for Cuf1 regulation of the SODs in response to Cu availability**. During Cu sufficiency (or excess) conditions, Cuf1 participates in activation of *SOD1* transcript expression through an unknown mechanism. Sod1 is localized to the cytosol, with a small but significant fraction in the mitochondrial IMS, and uses Cu as a cofactor (*blue circles*). In a Cuf1-independent manner, *SOD2* is transcribed and the protein is localized to the mitochondrial matrix through its amino-terminally encoded MIP (*yellow*), and uses Mn as a cofactor (*purple circles*). However, during Cu deficiency, Cuf1 recognizes downstream CuREs (CuRE1 and CuRE2) within the promoters of *SOD1* and *SOD2* (*blue boxes*, with CuRE sites denoted by numbering) resulting in the generation of alternative 5’-end truncated *SOD1* and *SOD2* transcripts. This results in a nonproductive transcript of *SOD1*, effectively repressing its expression. The shortened *SOD2* transcript encodes for the expression of two isoforms of Sod2 from a single mRNA: the canonical mitochondrially targeted isoform and a neo-cytosolic isoform lacking its encoded MIP. The role (if any) for *SOD1* CuRE3 is still not fully understood. CuRE, copper responsive element; IMS, intermembrane space; MIP, mitochondrial import peptide; SOD, superoxide dismutase.

Here, we demonstrate the Cu-sensing transcription factor Cuf1 represses Sod1 and generates an alternative isoform of Sod2 in response to Cu deficiency by altering the 5’ ends of the SOD transcripts (Fig. 10). This adaptation enables *C. neoformans* to efficiently colonize Cu-deficient niches within the host during infection and may additionally serve as a mechanism to spare the limited available Cu to be utilized for more essential cellular processes required for establishing infection. These results provide yet another critical link between the organism’s ability to sense and respond to limitations in Cu availability and cope with ROS stress. Additionally, our studies would imply an interesting connection between Cu and Mn sensing and regulation, which merits further investigation. Finally, the parallels of activation of a cytosolic Mn–SOD between the basidiomycete *C. neoformans* and the ascomycete *C. albicans*, with an evolutionary divergence of over ∼100,000 Mya, suggest a common mechanism among pathogenic fungi in counteracting ROS in Cu-deficient host niches through activation of alternative Mn-dependent antioxidant enzymes.

## Experimental procedures

### Generation of *C. neoformans* mutants

DNA was introduced into *C. neoformans* by biolistic transformation as previously described (81). Yeast peptone dextrose media, supplemented with 1.5% agar and 100 μg ml^−1^ of nourseothricin (NAT), 200 μg ml^−1^ of neomycin (G418) or 200 μg ml^−1^ of hygromycin B (HYG) were used for colony selection after biolistic transformation. Strains descriptions are in Table S3. Proper DNA integration was validated by PCR amplification of specific genomic loci and proper single copy integration at desired genomic loci validated by Southern blotting. Two independent isolates from each transformation were selected, evaluated, and saved as glycerol stocks.

### RNA isolation and qRT-PCR

*C. neoformans* overnight cultures grown in SC medium (MP Biomedicals) were diluted to OD_600_ of 0.2, recovered for 3 h at 30 °C with shaking at 200 rpm. After recovery period, cultures were supplemented as indicated. mRNA isolation and expression analysis were performed as previously described (42, 82). Primers for *SOD1*, *SOD2*, *MT1*, *CTR4*, and *GAPDH* for qRT-PCR are described in Table S4. Reactions were analyzed on a CFX384 C1000 ThermoCycler (BioRad), and C_T_ values were determined using the included CFX Maestro software (BioRad). Gene expression values were normalized to the housekeeping gene *GAPDH* and expression fold changes determined by the ΔΔC_T_ method. For all qRT-PCR studies, technical duplicates of independent biological triplicates (N = 3) were used for the analysis of mRNA expression changes.

### Chromatin immunoprecipitation

Overnight cultures of *C. neoformans cuf1*Δ cells complemented with Cuf1-FLAG were diluted to OD_600_ of 0.2 in SC media, recovered for 3 h at 30 °C, 200 rpm, and then supplemented with either 1 mM CuSO_4_ or 1 mM BCS for an additional 3 h. ChIP-qPCR analysis was performed as previously described (42). Specific primers used for detecting Cuf1 enrichment at *SOD1*, *SOD2*, and *TUB1* promoters by qPCR are described in Table S4. For all ChIP-qPCR studies, technical duplicates of independent biological triplicates (N = 3) were used for the analysis.

### Protein extraction and immunoblotting

*C. neoformans* cultures were harvested and washed three times in 1X PBS and resuspending in 3 ml/g cell pellet of lysis buffer (50 mM Hepes [pH 7.5], 140 mM NaCl, 1 mM EDTA, 1% Triton X-100, and 1X yeast-specific protease inhibitor [Sigma-Aldrich]). One milliliter resuspended cells were added to 2 ml screw cap Eppendorf tubes with added acid washed, autoclaved glass beads (425–600 μm, Sigma-Aldrich), and subjected to cell lysis by a Bead Ruptor 12 (Omni International) for 30 s on, 2 min off, ten cycles at 4 °C with chilling on ice in between cycles. Cell lysates were clarified by centrifugation at 5000*g* for 5 min, followed by centrifugation at 12,000*g* for 20 min. Clarified supernatants were assessed for protein content by BCA assay (Thermo Scientific) and proteins separated by 4 to 20% SDS-PAGE. Proteins were detected by immunoblotting using standard methods as previously described with the indicated antibodies.

### 5’-RACE

mRNA was isolated as described above and 5’ ends of *SOD1* and *SOD2* transcripts mapped by 5’-RACE using the FirstChoice RLM-RACE kit (Invitrogen) following manufacturer recommendations and final PCR products sequenced by Eton Bioscience. Gene specific primers for detecting *SOD1* and *SOD2* transcripts are listed in Table S4.

### Sod2-HA immunopurification

Fifty milliliter SC cultures of *SOD2HA C. neoformans* at a starting OD_600_ = 0.1 were supplemented with 5 μM CuSO_4_ or 1 mM BCS and grown for 16 h at 30 °C, 200 rpm. Cultures were harvested and washed three times in 1X PBS +5 mM EDTA. Cell pellets were resuspended in lysis buffer (10 mM Tris [pH 7.5], 20 mM NaCl, 1 mM EDTA, 1 mM PMSF, and 1X yeast protease inhibitor) and lysed using acid-washed glass beads in a Bead Ruptor 12 (30 s on, 2 min off, ten cycles). Lysates were then pulse sonicated on ice (15 s on, 45 s off, output setting 3, four cycles) and clarified by centrifugation at 20,000*g* for 15 min 150 μl HA-resin (ThermoFisher Scientific) prewashed in lysis buffer was added to clarified cell extracts and incubated on rotary platform overnight at 4 °C. Resin was pelleted and washed in 3 × 500 μl PBST, followed by another 500 μl PBS. Sod2-HA was eluted using 10 × 100 μl 3 M NaSCN. Elutions were pooled and concentrated using spin filters (EMD Millipore), then buffer exchanged into 10 mM Tris (pH 8.0) using desalting columns (ThermoFisher Scientific). Concentrations were measured using the theoretical extinction coefficient of 40,910 (M^−1^ cm^−1^) at A_280_ nm on a Nanodrop One^C^ (Thermo Scientific) as well as by BCA assay (Pierce). Protein purity was assessed by SDS-PAGE analysis and Coomassie staining.

### Mass spectrometry analysis of Sod2 amino terminus (Edman and MALDI)

Purified Sod2-HA samples were sent to the Molecular Structure Facility at the University of California, Davis for Edman sequencing analysis of amino termini. For MALDI-MS/MS analysis, purified Sod2-HA was loaded onto a 4 to 20% SDS-PAGE gel and stained with Coomassie blue. The purified band of Sod2-HA was excised and digested within the gel matrix with the endoproteinase GluC (New England Biolabs) following manufacturer’s guidelines, and peptide extracts were then frozen and lyophilized. The peptides were resuspended in 5 μl of 100:99:1 acetonitrile: water: trifluoroacetic acid immediately before spotting on the MALDI target. For MALDI analysis, the matrix solution consisted of alpha-cyano-4-hydroxycinnamic acid (Aldrich Chemical Co) saturating a solution of 1:1:0.01 acetonitrile: 25 mM ammonium citrate: trifuoroacetic acid. Approximately, 0.15 μl of peptide solution was spotted on the MALDI target immediately followed by 0.15 μl of the matrix solution. This combined solution was allowed to dry at room temperature. MALDI MS and MS/MS data were then acquired using the ABSCIEX 5800 TOF/TOF Mass Spectrometer.

### ICP-MS metal analysis of *C. neoformans* cells

Overnight cultures of *C. neoformans* in SC media were back diluted to an OD_600_ of 0.2 in 50 ml fresh SC media and recovered for 3 h at 30 °C, 200 rpm. Cultures were then supplemented with 5 μM CuSO_4_ or 1 mM BCS and incubated an additional 12 h, all in triplicate. Cultures were harvested at 3000*g* for 5 min and washed in 3× 5 ml PBS +5 mM EDTA, then 3× 5 ml ultratrace elemental analysis grade water (Fisher Scientific), and adjusted to a final cell concentration of 1 × 10^8^ cells/ml. 1 ml of cells (1 × 10^8^ cells total) were pelleted and frozen at −80 °C until ready for elemental analysis at the Oregon Health and Science University Elemental Analysis Core. Cells were digested with 100 μl of concentrated HNO_3_ (Thermo Fisher Scientific, trace metal grade) at 90 °C for 45 min. After digestion, 1000 μl of 1% HNO_3_ was added to each sample and metal concentrations measured by ICP-MS analysis using an Agilent 7700x equipped with an ASX 500 autosampler. Elements were measured in kinetic energy discrimination mode using He gas. Data were quantified using serial dilutions of a multielement standard (CEM 2, [VHG-SM70B-100]). Data were acquired in triplicates and averaged. A coefficient of variance was determined from frequent measurements of a sample containing ∼10 ppb of Mn and Cu, and 10 ppm of Ca. An internal standard (^45^Sc, ^72^Ge, ^209^Bi) continuously introduced with the sample was used to correct for detector fluctuations and to monitor plasma stability. Accuracy of the calibration curve was assessed by measuring NIST reference material (water, SRM 1643f) and found to within 97% to 102% for all elements. Accuracy of elemental recovery from digestion was accessed by digesting 4 μl of a 100 ppm calibration standard (“CEM2”, [VHG-SM70B-100] with 100 μl conc. HNO_3_ [trace metal grade, Thermo Fisher Scientific]) by the same method as the samples. Elemental concentrations of these controls were found to be within 95% to 104% of the expected concentration.

### *C. neoformans* subcellular fractionation

Overnight cultures of *C. neoformans* in SC media were back diluted to an OD_600_ of 0.2 in 50 ml fresh SC media and recovered for 3 h at 30 °C, 200 rpm. Cultures were then supplemented with 5 μM CuSO_4_ or 1 mM BCS and incubated an additional 12 h. Cultures were harvested at 3000*g* for 5 min and washed in 2× 5 ml PBS and pelleted as before. Pellets were resuspended in 2 ml/g wet weight cell pellet in DTT Buffer (100 mM Tris [pH 9.4], 10 mM DTT) and incubated at 30 °C with shaking at 70 rpm for 20 min. Cells were centrifuged 3000*g* for 5 min, and cell pellets resuspended in 6.5 ml/g wet weight cell pellet in lytic Buffer (100 mM sodium citrate [pH 5.5], 1.1 M sorbitol). Cells were centrifuged and resuspended as before in 6.5 ml/g lytic buffer. 25 mg/ml lysing enzymes from *Trichoderma harzianum* (Sigma Aldrich) were added to resuspended cells and incubated at 30 °C with gentle agitation at 70 rpm for 3 h. Generated spheroplasts were pelleted at 2200*g* for 8 min and gently washed in 7 ml/g wet weight cell pellet of ice-cold homogenization buffer (10 mM Tris [pH 7.4], 0.6 M sorbitol). Resuspended spheroplasts were pelleted and resuspended in homogenization Buffer as before but with added 1X fungal specific protease inhibitor cocktail (Sigma Aldrich). Spheroplasts were lysed by 20 strokes of Dounce homogenization on ice, then diluted 1:1 with cold homogenization buffer with added protease inhibitor cocktail. Debris was pelleted at 1500*g* for 5 min, and supernatants are collected and centrifuged once more at 3000*g* for 5 min, and supernatants were collected again. Clarified supernatants were then centrifuged 15,000*g* for 30 min. The postmitochondrial supernatants (cytosolic fractions) were collected from the pellets (crude mitochondria). Crude mitochondrial pellets were resuspended in 1.5 ml cold homogenization buffer and centrifuged once more at 15,000*g* for 30 min. Supernatants were combined with the cytosolic fraction, and mitochondrial pellets were resuspended in 200 μl of 10 mM Mops (pH 7.2) and briefly sonicated with a microprobe for 5 s ON and 10 s OFF with an output of 3. Cytosolic fractions were concentrated using centrifugal concentrators (EMD Millipore) with a 3 kDa MWCO to roughly 2 ml. Total protein content of lysed crude mitochondria and concentrated cytosolic fractions were quantified by BCA assay (Pierce). Samples were mixed with 4X Laemmli sample buffer (BioRad) under reducing conditions (with added 10% 2-mercaptoethanol) and boiled for 10 min at 90 °C. 30 μg and 10 μg of total protein extracts from cytosolic and mitochondrial fractions, respectively, were separated by 4 to 20% SDS-PAGE, transferred to nitrocellulose membranes, and immunoblotted with anti-HA (ab18181, monoclonal, Abcam), anti-ySod1 (a generous gift from T.V. O’Halloran), anti-GAPDH (ab9485, polyclonal, abcam), and anti-MTCO1 (ab110270, monoclonal, abcam) antibodies.

### *In vitro* SOD activity assay

*C. neoformans* cultures in SC media were supplemented with 1 mM BCS or 0.1 mM CuSO_4_ and incubated for 12 h at 30 °C, 200 rpm. Cultures were washed three times in 1X PBS and resuspended in lysis buffer (50 mM Tris [pH 7.4], 140 mM NaCl, 1 mM EDTA, and 1X yeast protease inhibitor [Sigma-Aldrich]) and lysed using acid-washed, autoclaved glass beads (425–600 μm, Sigma-Aldrich) in a Bead Ruptor 12 (30 s on, 2 min off, ten cycles). Lysates were then pulse sonicated on ice (15 s on, 45 s off, output setting 3, four cycles) and clarified by centrifugation at 20,000*g* for 15 min. Total protein concentrations of whole cell lysates were quantified by the BCA assay (Thermo Scientific), and total SOD activity and Sod2-specific activity were measured using the xanthine-xanthine oxidase and nitroblue tetrazolium assay as previously described (83).

### Disc diffusion assay

Overnight cultures of indicated *C. neoformans* strains were grown overnight in SC media. The following day cells were diluted to a final OD_600_ of 0.1 in 10 ml of top agar (SC + 0.7% agar) cooled to 40 °C and supplemented with 1 mM BCS or 0.1 mM CuSO_4_. Cells suspended in top agar were poured onto SC agar plates containing identical concentrations of either CuSO_4_ or BCS and allowed to solidify. Filter discs were spotted with 10 μl of the indicated concentrations of menadione (resuspended in ethanol) or hydrogen peroxide (diluted in water) and set in quadrants around the plates. Plates were incubated at 30 °C for 3 to 4 days and photographed for qualitative comparison.

### Mouse infection experiments

Inbred male and female A/J mice (Jackson Laboratories) aged 6 to 8 weeks were used for virulence experiments. Purchased mice were acclimated for 1 week housed at the Duke Vivarium. For infections all *C. neoformans* strains were grown in SC media overnight and washed 3 times with sterile 1X PBS. Cell concentrations were measured using a cell counter (BioRad) and resuspended in 1X PBS to 3333 cells/ml. One hundred and fifty microliter of cell suspension (500 cells) or 150 μl of 1X PBS (mock control) was administered to mice anesthetized with isoflurane by retro-orbital injection. Aliquots of inoculum from each strain was also plated on SC agar plates for quantitation of colony forming units as secondary validation of cell numbers. Mouse infection experiments were performed in compliance with all ethical regulations. The mouse protocol (A257-18-11) was revised and approved by the Duke University Institutional Animal Care and Use Committee (DUIACUC). Experimental endpoints were considered a 15% reduction in initial body weight.

### Fungal burden assays

Mice were infected as described above and monitored for 3 or 7 days postinfection. At the indicated timepoint, mice were humanely euthanized. Before organ harvesting, mice were perfused with 10 ml 1X PBS by cardiac perfusion. The wet weight of all harvested organs was recorded and placed in 15 ml round bottom tubes containing 5 ml 1X PBS and homogenized using a tissue homogenizer (Omni International). Serial dilutions of homogenized organs in 1X PBS were plated on SC agar plates and incubated for 3 days at 30 °C and CFUs enumerated to assess tissue specific fungal burden.

### Statistical analysis

For all data, error bars represent the standard deviations of results from a number of biological replicates (N), as indicated in figure legends. The statistical tests chosen for each experiment are indicated in figure legends. Asterisks in figures correspond to statistical significance as indicated in the figure legends: ∗∗∗*p* < 0.005; ∗∗*p* = 0.005 to *p* < 0.01; ∗*p* = 0.01 to *p* < 0.05; n.s. (not significant), *p* > 0.05.

## Data availability

The data that support the findings of this study are available from the corresponding author upon reasonable request.

## Supporting information

This article contains supporting information.

## Conflict of interests

The authors declare that they have no conflicts of interest with the contents of this article.
