# Supplementary material for: Transcription factor–driven alternative localization of Cryptococcus neoformans superoxide dismutase
Source: J Biol Chem. 2021 Feb 7;296:100391. doi: 10.1016/j.jbc.2021.100391 (PMC7961099; doi:10.1016/j.jbc.2021.100391)
Supplement: Supplemental Figures S1–S9 and Tables S1–S4 [file mmc1.pdf]

Supporting Information

Transcription factor-driven alternative localization of *Cryptococcus neoformans* superoxide dismutase

Aaron D. Smith[1,#a], Sarela Garcia-Santamarina[1,#b], Martina Ralle[2], David R. Loiselle[1], Timothy A. Haystead[1], and Dennis J. Thiele[1,3,4*#c]

[1]Department of Pharmacology and Cancer Biology, Duke University, Durham, North Carolina, United States of America

[2]Department of Molecular and Medical Genetics, Oregon Health and Science University, Portland, Oregon, United States of America

[3]Department of Biochemistry, Duke University, Durham, North Carolina, United States of America

[4]Department of Molecular Genetics and Microbiology, Duke University, Durham, North Carolina, United States of America

[#a]Present address: College of Sciences, George Mason University, Fairfax, Virginia, United States of America

[#b]Present address: The European Molecular Biology Laboratory, Heidelberg, Germany

[#c]Present address: Sisu Pharma, Inc.

*Corresponding author: Dennis J. Thiele

E-mail: dthiele@sisupharma.com (DJT)

Supporting Information:

Figures S1 – S9

Tables S1 – S4

a

```
CnSod1          -MVKAVVVLKGESYVHGTVCFTQESENAPVCITGEIKDMDADAKRGMHVHEFGDNTNGCT
ScSod1          -MVQAVAVLKGDAGVSGVVKFEQASESEPTTVSYEIAGNSPNAERGFHIHEFGDATNGCV
CaSod1          -MVKAVAVVRGDSKVQGTVHFEQESESAPTTISWEIEGNDPNALRGFHIHQFGDNTNGCT
HsSod1          MATKAVCVLKGDGPVQGIINFEQKESNGPVKVWGSIKGLT-EGLHGFHVHEFGDNTAGCT
                  .:** *:.*:. * * : * * ... *. :  .* .    :. .*:*:*:*** * **.

CnSod1          SAGPHYNPFKKHHGAPTDSERHVGDLGNIQTNSCGAAQLDFSDKIISLYGPHSIIGRSLV
ScSod1          SAGPHFNPFKKTHGAPTDEVRHVGDMGNVKTDENGVAKGSFKDSLIKLIGPTSVVGRSVV
CaSod1          SAGPHFNPFGKQHGAPEDDERHVGDLGNISTDGNGVAKGTKQDLLIKLIGKDSILGRTIV
HsSod1          SAGPHFNPLSRKHGGPKDEERHVGDLGNVTADKDGVADVSIEDSVISLSGDHCIIGRTLV
                *****:**: . **.* *. *****:**: ::  *.*.    .* :*.* *  .::**::*

CnSod1          VHASTDDLGKGGNEESLKTGNAGARLACGVIGIST
ScSod1          IHAGQDDLGKGDTEESLKTGNAGPRPACGVIGLTN
CaSod1          VHAGTDDYGKGGFEDSKTTGHAGARPACGVIGLTQ
HsSod1          VHEKADDLGKGGNEESTKTGNAGSRLACGVIGIAQ
                :*   ** ***. *:* .**:**.* ******::
```

b

```
CnSod2          MITAIT-------RTALPRATLRTSLATMSTIRAKHTLPPLPYAYDALEPSISAEIMNLH
HsSod2          MLSR---------AVCGTSRQLAPVLGY-LGSRQKHSLPDLPYDYGALEPHINAQIMQLH
ScSod2          MFAKTA-------AANLTKKGGLSLLST-TARRTKVTLPDLKWDFGALEPYISGQINELH
CaSod2          MFSIRSSSRVLLKASSATTRATLNAAASKTFTRSKYSLPELDYEFSATEPYISGQINEIH
                *::               .         .      * * :** * : :.* ** *..:* ::*

CnSod2          HTKHHQTYVNGLNAAEESLQKAS-------ADGDFKTAISLQPALKFNGGGHINHSLFWK
HsSod2          HSKHHAAYVNNLNVTEEKYQEAL-------AKGDVTAQIALQPALKFNGGGHINHSIFWT
ScSod2          YTKHHQTYVNGFNTAVDQFQELSDLLAKEPSPANARKMIAIQQNIKFHGGGFTNHCLFWE
CaSod2          YTKHHQTYVNNLNASIEQAVEAK-------SKGEVKKLVALQKAINFNGGGYLNHCLWWK
                ::*** :***.:*.: :.  :          : .:    :::*  ::*:***. **.::*

CnSod2          NLAPTGSAQVKVPT-SGVFYDQVQADFGGFENLKKEMNAKTAAIQGSGWGWLGYNKATK-
HsSod2          NLSP--NGGGEPK---GELLEAIKRDFGSFDKFKEKLTAASVGVQGSGWGWLGFNKERG-
ScSod2          NLAPESQGGGEPPT--GALAKAIDEQFGSLDELIKLTNTKLAGVQGSGWAFIVKNLSNGG
CaSod2          NLAPVSQGGGQPPSEDSKLGKQIVKQFGSLDKLIEITNGKLAGIQGSGWAFIVKNKANGD
                **:*   ..  :      . : . :  :**.:::: :  .    ..:*****.::   *

CnSod2          KLEIVTTPNQDPLLSH---VPIIGIDIWEHAFYLQYKNVKPDYLNAIWNVINYEEAESRL
HsSod2          HLQIAACPNQDPLQGTTGLIPLLGIDVWEHAYYLQYKNVRPDYLKAIWNVINWENVTERY
ScSod2          KLDVVQTYNQDTVTGP--LVPLVAIDAWEHAYYLQYQNKKADYFKAIWNVVNWKEASRRF
CaSod2          TIDVITTANQDTVTDPN-LVPLIAIDAWEHAYYLQYQNVKADYFKNLWHVINWKEAERRF
                 :::     ***.: .     :*::.** ****:****:* ..**:: :*:*:*:::.  *

CnSod2          KAAQ-
HsSod2          MACKK
ScSod2          DAGKI
CaSod2          EF---
```

Figure S1. Multiple sequence alignment of Sod1 and Sod2 proteins

Multiple sequence alignments of Sod1 (**a**) and Sod2 (**b**) reveal the highly conserved nature of the SOD enzymes and conservation of metal coordinating ligands that comprise the enzyme active sites across distantly related species. In (**a**) light blue highlights the Cu-coordinating ligands, light grey highlights the Zn-coordinating ligands, while the yellow highlights a shared bridging histidine residue between the Zn and Cu in Sod1. In (**b**) magenta highlights the conserved Mn-coordinating ligands of Sod2. Cn – *C. neoformans*; Sc – *S. cerevisiae*; Ca – *C. albicans*; Hs – *Homo sapiens*.

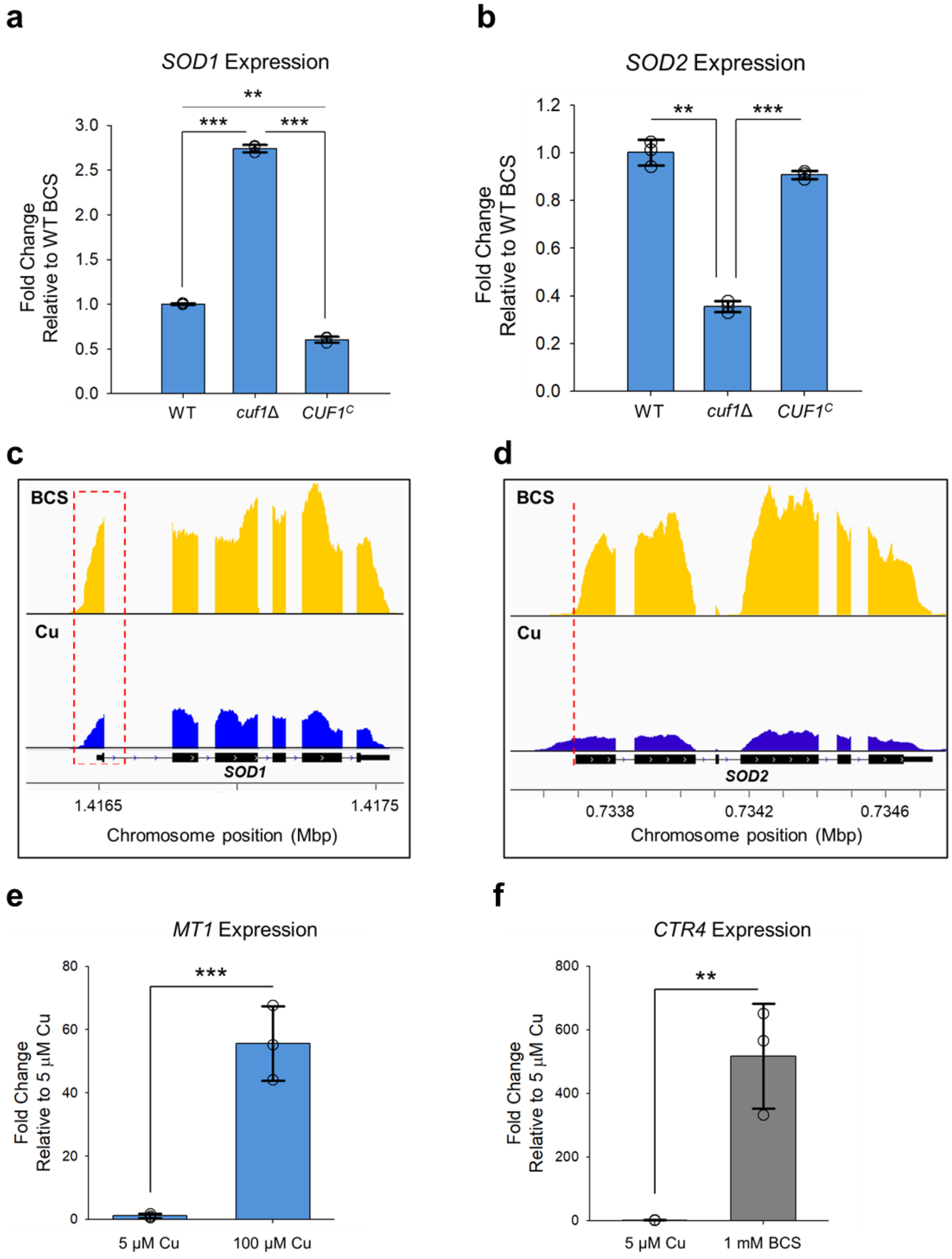

Figure S2. The differential regulation of *SOD1* and *SOD2* is Cuf1-dependent.
(**a**) qRT-PCR analysis of *SOD1* transcript levels in the WT, *cuf1Δ*, and $CUF1^{C}$ strains when treated with 1 mM BCS or 0.3 mM $CuSO_4$. Expression levels normalized to the WT strain. N=3, 2-tailed t-test. (**b**) qRT-PCR analysis of *SOD2* transcript levels as in (**a**). N=3, 2-tailed t-test. mRNA-seq analysis of (**c**) *SOD1* and (**d**) *SOD2* in the *cuf1*Δ strain in cells treated with BCS (yellow) vs Cu (blue). Region of 5'-truncation observed in the WT strain is indicated by the red box and red dashed line for *SOD1* and *SOD2*, respectively. Sequencing reads were aligned to the *C. neoformans* H99 genome and visualized using the integrative genome viewer (IGV) (84). X-axis representative of chromosomal location (in Mbp). (**e**) qRT-PCR analysis of *MT1* expression in cells treated with 0.1 mM $CuSO_4$ vs 5 µM $CuSO_4$. N=3, 2-tailed t-test. (**f**) qRT-PCR analysis of *CTR4* expression in cells treated with 1 mM BCS vs 5 µM $CuSO_4$. N=3, 2-tailed t-test. ** $p < 0.01$, *** $p < 0.005$.

a

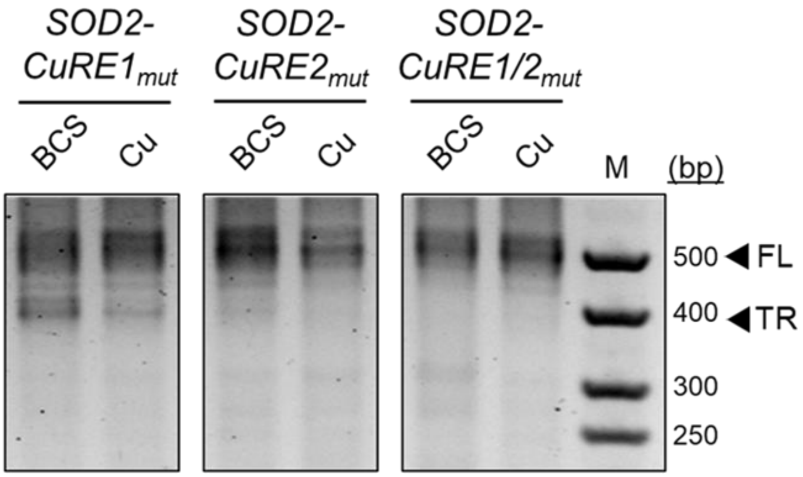

b

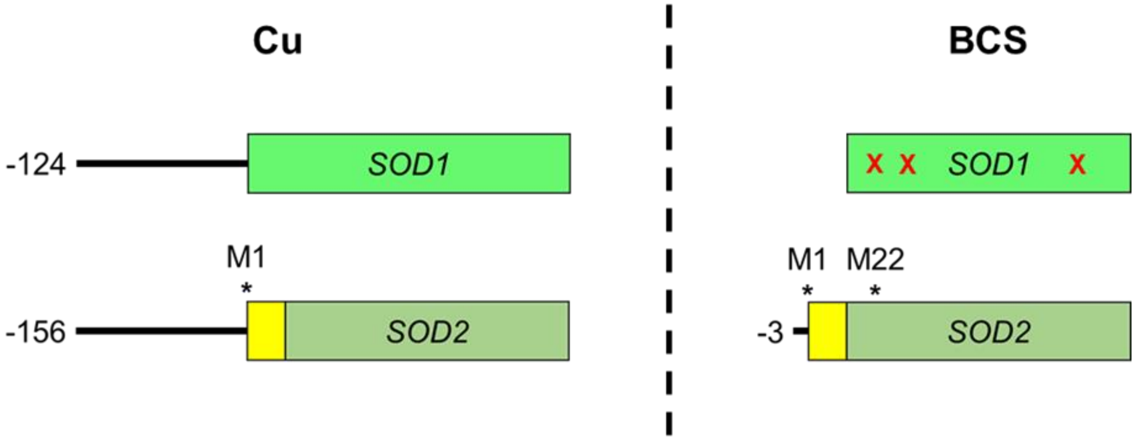

Figure S3. Both CuRE sequences and Cuf1 are required for maximal induction of the 5'-shortened *SOD1* and *SOD2* transcripts.
(**a**) Visualization of final 5'-RACE PCR products of *SOD1* and *SOD2* from the indicated strains treated with 1 mM BCS or 5 µM $CuSO_4$. Products were separated by agarose gel electrophoresis and visualized by ethidium bromide staining. **FL** – full length, **TR** – truncated or shortened transcript. (**b**) Schematic representation of the sequenced 5'-ends of the *SOD1* and *SOD2* transcripts of Cu sufficient or Cu deficient cells. Numbers represent the distance of the 5'-UTR to the translation initiation codon (+1). For the shortened *SOD1* transcript, the red "x" represents premature stop codons as a result of frameshifting. For the *SOD2* transcript, the yellow box represents the amino-terminally encoded mitochondrial import peptide and the locations of the canonically initiating codon (M1) and the downstream alternative initiating codon (M22) are indicated with asterisks.

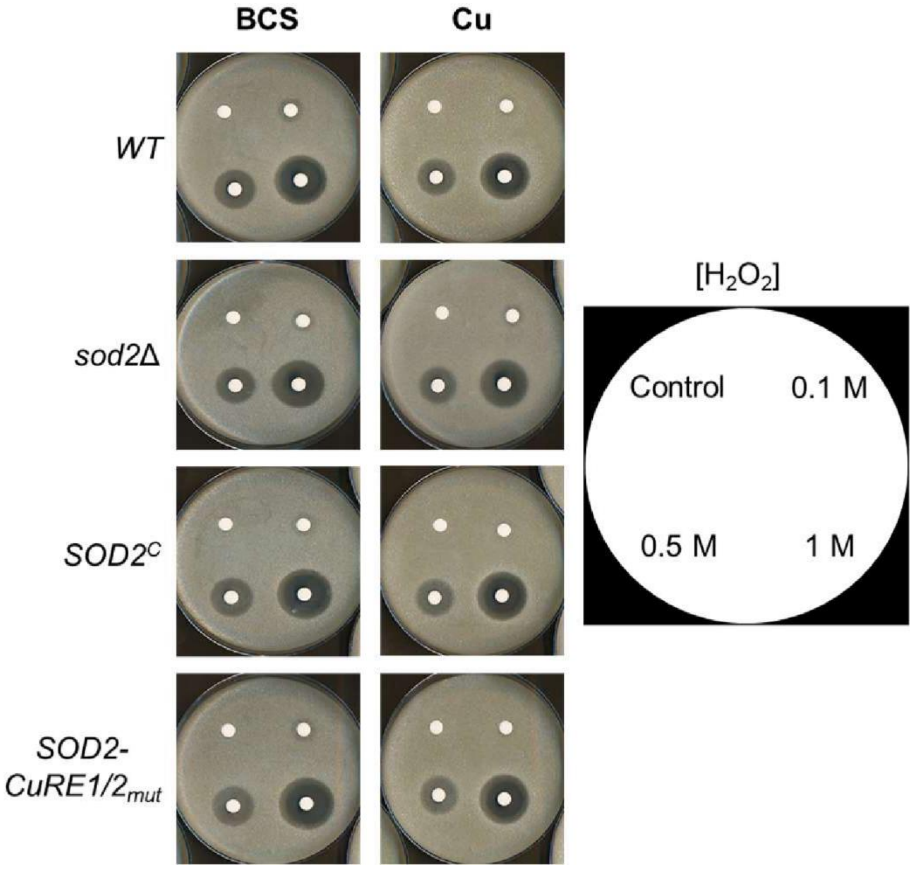

Figure S4. Cytosolic Sod2 protects cells specifically from superoxide stress during Cu limitation. *In vivo* cellular hydrogen peroxide sensitivities as tested by disc diffusion assays in the indicated strains and in the presence of 1 mM BCS or 0.1 mM $CuSO_4$. Cells were challenged by spotting increasing concentrations of hydrogen peroxide on filter discs and allowing cells to incubate at 30°C for 3 days. Hydrogen peroxide concentrations are indicated on the key to the right.

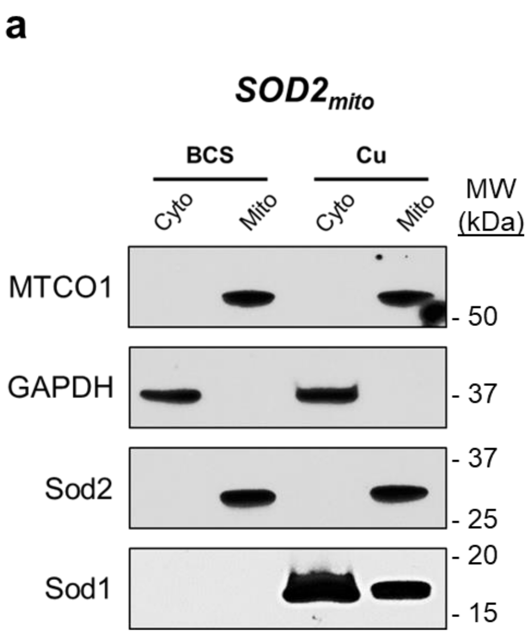

Figure S5. ***SOD2**$_{mito}$* **and** ***SOD2**$_{cyto}$* **exclusively express Sod2 in the mitochondria and cytosol, respectively.**
Subcellular fractionation of (**a**) *SOD2*$_{mito}$ and (**b**) *SOD2*$_{cyto}$ strains constitutively expressing Sod2 under transcriptional control of the actin (ACT1) promoter under Cu deficient (1 mM BCS) or Cu sufficient (5 μM $CuSO_4$) conditions. Sod1 and Sod2 localization were visualized by Western blotting. Control proteins specific to cytosol (GAPDH – Glyceraldehyde 3-phosphate dehydrogenase) and mitochondria (MTCO1 – Mitochondrial COX1 subunit) were included to validate fractionation efficacy.

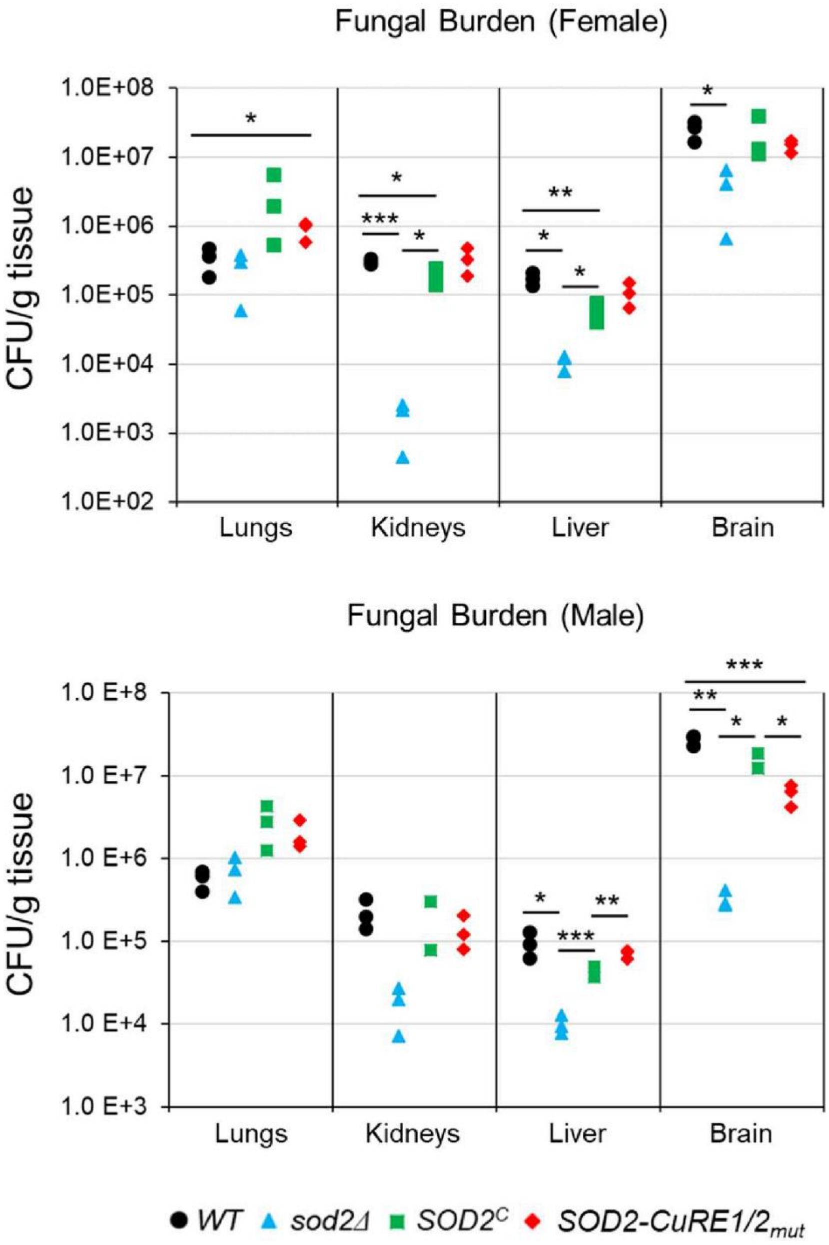

Figure S6. Fungal burden in mice at 7 days post infection
C. neoformans CFU analysis of the indicated strains from organs harvested from both male and female A/J mice 7 days post infection. Statistical differences were assessed across groups by the nonparametric Kruskal-Wallis one-way analysis of variance. F-test for variances was used between sample groups and significance determined by a 2-tailed t-test. N=3. * $p < 0.05$, ** $p < 0.01$, *** $p < 0.005$.

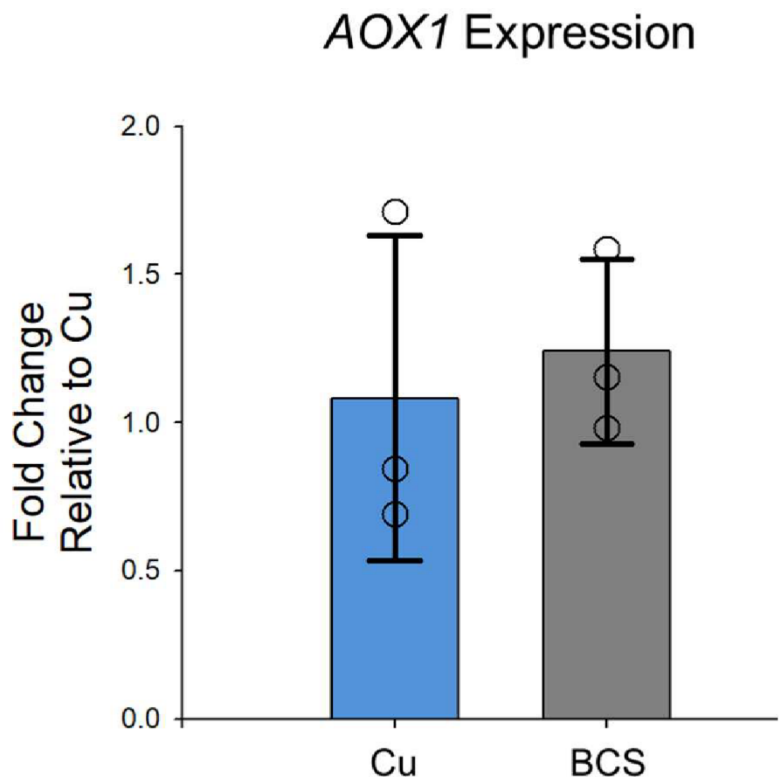

Figure S7. ***AOX1*** **expression in Cu limited** ***C. neoformans*** **cells**
qRT-PCR analysis of *C. neoformans AOX1* in cells supplemented with 5 µM $CuSO_4$ or 1 mM BCS. N=3, 2-tailed t-test.

a

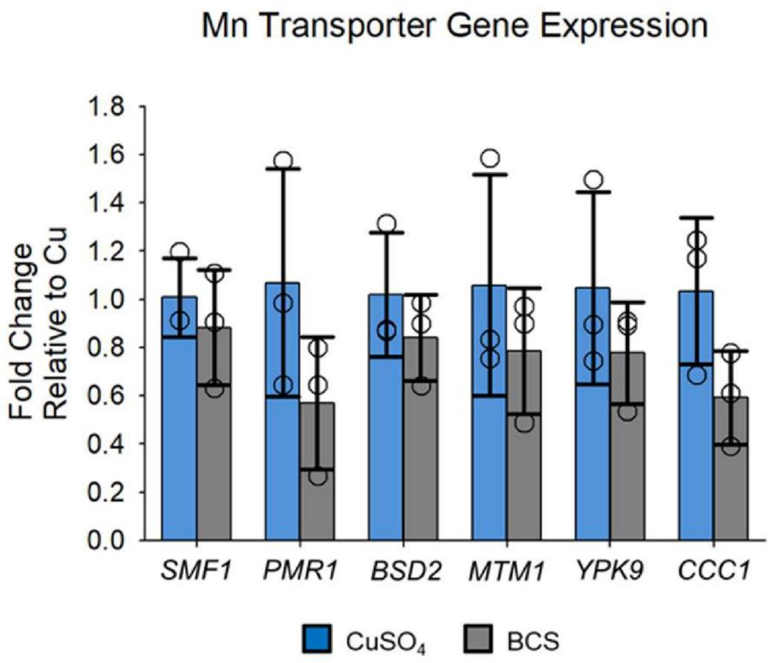

b

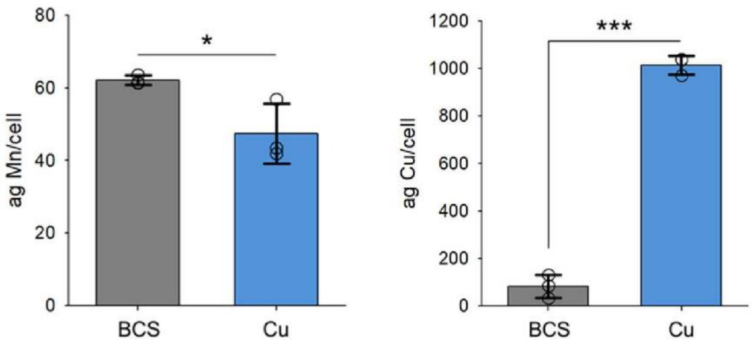

Figure S8. Cellular Mn homeostasis in Cu limited *C. neoformans* cells
(**a**) qRT-PCR analysis of known Mn transporter genes in cells supplemented with 5 µM $CuSO_4$ or 1 mM BCS. N=3, 2-tailed t-test. (**b**) ICP-MS metal analysis of Mn (left) and Cu (right) in whole cells supplemented with 1 mM BCS or 5 µM $CuSO_4$. N=3, 2-tailed t-test.

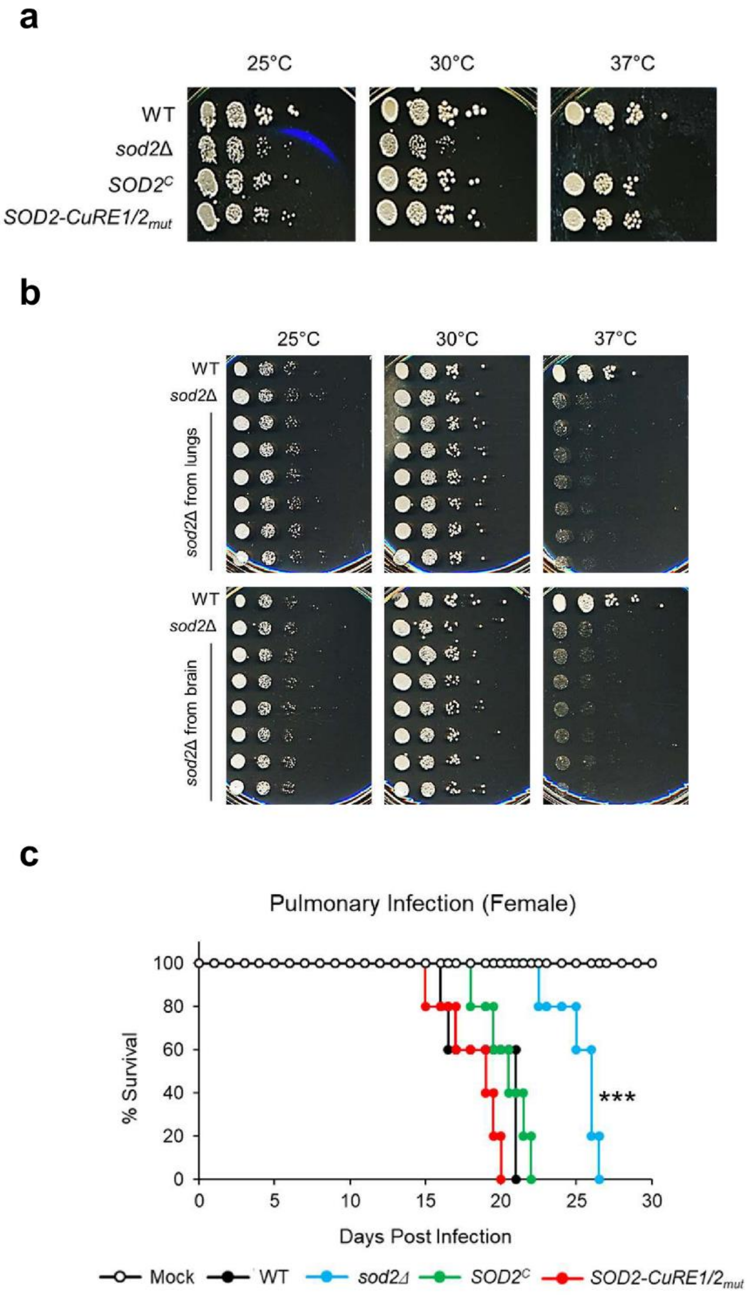

Figure S9. Validation the *sod2Δ* strain has a temperature sensitive phenotype prior to and after mouse infection

(**a**) Strains with the indicated genotypes were prepared as inoculum for the retro-orbital murine infection assays, serially diluted in 1X PBS, then spotted onto SC agar plates and incubated at 25°C, 30°C, or 37°C for 3-4 days. (**b**) WT and *sod2Δ* cells from glycerol stocks were grown overnight and serially diluted in 1X PBS for spotting assays. Additionally, randomly picked *sod2Δ* colonies from the CFU analysis at 7 days post infection were grown overnight and serially diluted in 1X PBS for spotting assays. Random colonies were selected from both lung and brain homogenates. Plates were incubated at 25°C, 30°C, or 37°C for 3-4 days. (**c**) Pulmonary infection with 5,000 cells of WT or the *sod2Δ* strains administered to female A/J mice. The survival average of mice infected with the wild-type (WT) was compared to the survival average of mice infected with any of the mutants by the long rank statistical test. Statistical differences were found between mice infected with wild-type *vs* mice infected with mutants. N=5. * $p < 0.05$, ** $p < 0.01$, *** $p < 0.005$

	CuRE Site	nt Position*	Location	Motif Sequence
SOD1	CuRE1	-114	5'-UTR	AAAGGCCTCA
	CuRE2	-91	5'-UTR	ATACGCCTCA
	CuRE3	+42	Intron 1	ATATCGCTCA
SOD2	CuRE1	-143	5'-UTR	TAAAGCCTCA
	CuRE2	-91	5'-UTR	AATTGCCTCA

*Relative to translation start site (**A**TG)

Table S1. CuRE sequences of *C. neoformans SOD1* and *SOD2*
Nucleotide positioning is relative to the start of translation and context as to the location of the CuRE sequence within the genome is described.

	BCS		Cu	
Cycle #	Sequence	pmol	Sequence	pmol
1	Lys	24	Lys	11.7
2	His	13	His	5.5
3	Thr	14	Thr	7.4
4	Leu	18	Leu	8.3
5	Pro	14	Pro	7.9
6	Pro	-	Pro	-
7	Leu	13	Leu	6.5
8	Pro	8	Pro	4.2

Table S2. Sequencing results from Edman degradation of purified Sod2
Identification of amino-terminal residues of immuno-purified Sod2 from successive cycles of Edman degradation.

Table S3. List of strains used in this study

Strain ID	Strain	Genotype	Description	Source
DTY758	WT	Wild type *C. neoformans* H99	Wild type H99	J. Heitman lab, Duke University
DTY761	*cuf1* Δ	*cuf1::NEO*	*CUF1* deletion by replacement with NEO cassette	(43)
DTY762	*CUF1* ^{C}	*cuf1::NEO* , *CUF1::NAT*	DTY761 complemented with WT copy of *CUF1*	(43)
DTY982	*CUF1CFLAG*	*cuf1::NEO* , *CUF1CFLAG::NAT*	DTY761 complemented with C-terminal FLAG tagged *CUF1*	(26)
SG96	*SOD2-HA*	*SOD2HA* ::*NEO*	*SOD2* with C-terminal HA-epitope tag at endogenous genomic locus	This study
AS013	*sod2* Δ	*sod2* ::*NAT*	*SOD2* deletion by replacement with NAT cassette	This study
AS015	*SOD2* ^{C}	*sod2* ::*NAT* , *SOD2HA* ::*HYG*	*sod2* Δ complemented with wild type *SOD2* at safe haven	This study
AS017	*SOD2-CuRE1* $_{mut}$	*sod2* ::*NAT* , *SOD2HA-CuRE1* $_{mut}$ ::*HYG*	*sod2* Δ complemented with a *SOD2* CuRE1 promoter mutant at safe haven	This study
AS018	*SOD2-CuRE2* $_{mut}$	*sod2* ::*NAT* , *SOD2HA-CuRE2* $_{mut}$ ::*HYG*	*sod2* Δ complemented with a *SOD2* CuRE2 promoter mutant at safe haven	This study
AS019	*SOD2-CuRE1/2* $_{mut}$	*sod2* ::*NAT* , *SOD2HA-CuRE1/2* $_{mut}$ ::*HYG*	*sod2* Δ complemented with a *SOD2* CuRE1/2 double promoter mutant at safe haven	This study
AS021	*SOD2* $_{cyto}$	*sod2* ::*NAT* , *pACT1-SOD2HA(M22)* ::*HYG*	*sod2* Δ complemented with *SOD2* starting at downstream initiation codon (Met22) under constitutive actin (*ACT1*) promoter at safe haven	This study
AS023	*SOD2* $_{mito}$	*sod2* ::*NAT* , *pACT1-SOD2HA(M1)* ::*HYG*	*sod2* Δ complemented with *SOD2* starting at first initiation codon (Met1) under constitutive actin (*ACT1*) promoter at safe haven	This study
AS029	*SOD2* ^{C} , *CUF1CFLAG*	*sod2::NAT, SOD2HA::HYG, CUF1CFLAG::NEO*	*SOD2* ^{C} strain with C-terminal FLAG tagged *CUF1* at endogenous locus	This study
AS030	*CuRE1/2* $_{mut}$, *CUF1CFLAG*	*sod2::NAT, SOD2HA-CuRE1/2* $_{mut}$ *::HYG, CUF1CFLAG::NEO*	*CuRE1/2* $_{mut}$ strain with C-terminal FLAG tagged *CUF1* at endogenous locus	This study
AS033	*SOD1-FLAG*	*SOD1FLAG* ::*HYG*	*SOD1* with a C-terminal FLAG-epitope tag at endogenous genomic locus	This study
AS035	*sod1* Δ	*sod1::NEO*	*SOD1* deletion by replacement with NEO cassette	This study
AS037	*SOD1* ^{C}	*sod1::NEO, SOD1FLAG::HYG*	*sod1* Δ complemented with wild type *SOD1* at safe haven	This study
AS039	*SOD1-CuRE1/2* $_{mut}$	*sod1::NEO, SOD1FLAG-CuRE1/2* $_{mut}$ *::HYG*	*sod1* Δ complemented with a *SOD1* CuRE1/2 double promoter mutant at safe haven	This study
AS041	*SOD1-CuRE3* $_{mut}$	*sod1::NEO, SOD1FLAG-CuRE3* $_{mut}$ *::HYG*	*sod1* Δ complemented with a *SOD1* CuRE3 promoter mutant at safe haven	This study
AS043	*SOD1-CuRE1/2/3* $_{mut}$	*sod1::NEO, SOD1FLAG-CuRE1/2/3* $_{mut}$ *::HYG*	*sod1* Δ complemented with a *SOD1* CuRE1/2/3 triple promoter mutant at safe haven	This study

Table S4. List of primers used in this study

Primer ID	Sequence (5' -> 3')	Description	Experiment
AS082	TATAAAGCTTGAGCAAGGTTCTTCCAGAAGA	SOD2 gene specific	5'-RACE primer
AS085	ATATAAGCTTCCGTAGAGGGAGATGATCTT	SOD1 gene specific	5'-RACE primer
AS115	GCCTTCGTAATAAATGAAATTGTAGAGCCGCA GCCCCATC	SOD2 CuRE1 SDM F	Mutagenesis primer
AS116	GATGGGGCTGCGGCTCTACAATTTCATTTATT ACGAAGGC	SOD2 CuRE1 SDM R	Mutagenesis primer
AS117	CGTCTCTTGCTTCTAGTTATTGTACCAACCGC ACAGTCC	SOD2 CuRE2 SDM F	Mutagenesis primer
AS118	GGACTGTGCGGTTGGTACAATAACTAGAAGCA AGAGACG	SOD2 CuRE2 SDM R	Mutagenesis primer
AS291	TAAGCTTATGAATGAAAGTCGG	MT1 F	ChIP-qPCR
AS292	CAGCTTCTGGATTGCTGTT	MT1 R	ChIP-qPCR
AS293	GATTGGCATCAATCTGAGCA	CTR4 F	ChIP-qPCR
AS294	CATCTAGCGGGAAGGTTGTT	CTR4 R	ChIP-qPCR
AS295	TGAGTGAAAGTGGCTCATCG	TUB1 F	ChIP-qPCR
AS296	AGCAAGCCAAAAACAACACC	TUB1 R	ChIP-qPCR
AS297	CCACACTCGGCACTATCG	SOD1 F	ChIP-qPCR
AS298	GAGGAAGATATGGATGAGAG	SOD1 R	ChIP-qPCR
AS301	GGTGATGATGGGAATGACG	SOD2 F	ChIP-qPCR
AS303	GGTGTATGAGATACTGTGG	SOD2 R	ChIP-qPCR
ASq121	GGGTAACTGCGAATGCAAAG	MT1 F	qRT-PCR
ASq122	ACAAGCCTCACCAGATCCAC	MT1 R	qRT-PCR
ASq123	GCTGAGTATGGGAAGGATGG	CTR4 F	qRT-PCR
ASq124	ACCACGAAGGATTTGGTGAG	CTR4 R	qRT-PCR
ASq127	CGAGTCCCTACCTCTGATG	GAPDH F	qRT-PCR
ASq128	TCGGAAGCCTTCTTGATGAC	GAPDH R	qRT-PCR
ASq131	CTCGACTTTTCCGACAAGATC	SOD1 F	qRT-PCR
ASq132	TTGCCGAGGTCGTCAGTAC	SOD1 R	qRT-PCR
ASq145	GGACCCTCTTTTGTCTCACG	SOD2 F	qRT-PCR
ASq146	TGAGGTAGTCGGGCTTAACG	SOD2 R	qRT-PCR
Act1-v1 F	CCACCCACTGCCCAAGTAAA	ACT1 F	qRT-PCR
Act1-v1 R	CCTTGCACATACCAGAGCCA	ACT1 R	qRT-PCR
Bsd2-v1 F	GGTCCCGATGCTGACAATGA	BSD2 F	qRT-PCR
Bsd2-v1 R	GGTGGCGGCAACGTATAATC	BSD2 R	qRT-PCR
Ccc1-v1 F	GTGGCGGGCAATGTTGTAAG	CCC1 F	qRT-PCR
Ccc1-v1 R	TGTGAGACCATCGGAAAGGC	CCC1 R	qRT-PCR
Mtm1-v1 F	AGCTCTTCCTATCCCCGGAA	MTM1 F	qRT-PCR
Mtm1-v1 R	GATGGGGCCTTTGGATTGGA	MTM1 R	qRT-PCR
Pmr1-v1 F	TACTCCACCACCCTCAGGC	PMR1 F	qRT-PCR
Pmr1-v1 R	ATGACCGTTTCGAACAGCCC	PMR1 R	qRT-PCR
Smf1-v1 F	TGTTATGCGCGGGTCAAAGT	SMF1 F	qRT-PCR
Smf1-v1 R	ATGAGCCGACGAAGGAATGG	SMF1 R	qRT-PCR
Ypk9-v1 F	CATTGCGAAGGATGAGCGTG	YPK9 F	qRT-PCR
Ypk9-v1 R	TGGGGAAATGATGAGGGGTC	YPK9 R	qRT-PCR
